# Inflammation and Colorectal Cancer Pathogenesis: Molecular, Immunological, and Environmental Features for Therapy Response and Resistances

**DOI:** 10.3390/ijms27104299

**Published:** 2026-05-12

**Authors:** Barbara Seliger, Rene Mantke, Norbert Naß, Werner Dammermann, Soeren Ocvirk, Janine Kah, Thomas Kalinski, Chiara Massa

**Affiliations:** 1 Institute of Translational Immunology, Brandenburg Medical School “Theodor Fontane”, 14770 Brandenburg an der Havel, Germany; chiara.massa@mhb-fontane.de; 2 Faculty of Health Sciences Brandenburg, Brandenburg Medical School “Theodor Fontane”, 14770 Brandenburg an der Havel, Germany; werner.dammermann@mhb-fontane.de (W.D.); janine.kah@mhb-fontane.de (J.K.); 3 Centre for Translational Medicine, Brandenburg Medical School “Theodor Fontane”, 14770 Brandenburg an der Havel, Germany; norbert.nass@mhb-fontane.de; 4 Section of Immunopathology, Institute of Pathology, Martin-Luther-University Halle-Wittenberg, 06112 Halle (Saale), Germany; 5 Fraunhofer Institute for Cell Therapy and Immunology, 04103 Leipzig, Germany; 6 Clinic for General and Visceral Surgery, University Hospital Brandenburg, Brandenburg Medical School “Theodor Fontane”, 14770 Brandenburg an der Havel, Germany; mantke.mhb@uk-brandenburg.de; 7 Department of Pathology, University Hospital Brandenburg, Brandenburg Medical School “Theodor Fontane”, 14770 Brandenburg an der Havel, Germany; thomas.kalinski@uk-brandenburg.de; 8 Department of Gastroenterology, University Hospital Brandenburg, Brandenburg Medical School “Theodor Fontane”, 14770 Brandenburg an der Havel, Germany; 9 Gnotobiology Research Unit, German Institute of Human Nutrition Potsdam-Rehbruecke, 14558 Nuthetal, Germany; soeren.ocvirk@dife.de; 10 Research Group WESTGUT, ZIEL, Institute for Food & Health, Technical University of Munich, 85354 Freising, Germany; 11 Department of Medicine, University Medical Center Hamburg-Eppendorf, 20251 Hamburg, Germany

**Keywords:** colorectal cancer, tumor microenvironment, therapy, biomarkers, risk factors

## Abstract

Colorectal cancer (CRC) is a significant global health concern, ranking as the third most frequently diagnosed cancer and the second leading cause of cancer-related deaths. Advances in screening, such as the implementation of liquid biopsies (LB), have improved early detection, thus enhancing survival rates. This review summarizes the multifaceted nature of CRC, focusing on its genetic background, the complex tumor microenvironment, and the influence of gut microbiota, nutrition, and metabolic alterations. The development of CRC is influenced by various risk factors, including age, genetics, chronic diseases, and lifestyle choices. The genetic heterogeneity of CRC defines distinct molecular subtypes, characterized by different treatment responses and patient prognoses. Chronic inflammation and dysbiosis in the gut microbiota further contribute to CRC pathogenesis. In addition, nutritional factors play a crucial role in CRC, affecting carcinogenesis and treatment efficacy through direct interaction with the immune system and microbiome. Current therapeutic strategies include surgical interventions, chemo- and radiotherapy, targeted therapies, immunotherapy as well as dietary interventions, and microbiome modulation, highlighting the challenges posed by tumor heterogeneity and treatment resistance. In sum, a comprehensive understanding of CRC’s intrinsic and extrinsic drivers, including genetic, metabolic, and dietary influences, is essential for developing personalized treatment strategies and improving patient outcomes.

## 1. Introduction

Colorectal cancer (CRC) is the third most commonly diagnosed cancer worldwide, accounting for approx 10% of all cancer diagnoses and the second leading cause of cancer-related death [1]. During recent decades, comprehensive screening programs have significantly contributed to the early detection of this disease, leading to a higher overall survival (OS) rate of CRC patients. While the five-year survival rate for patients with advanced-stage CRC is about 14%, it rises to approx 90% for early-stage CRC. In order to further improve the outcome of CRC patients, a deep understanding of the intrinsic characteristics of the malignant cells and how they interact with the host to form a supportive tumor microenvironment (TME) that can also impair therapeutic treatment is needed. In the next paragraphs, we will highlight the different causes of CRC development, the current strategies for patients’ screening and treatment, as well as new therapeutic approaches targeting metabolism at the patient and cellular levels.

## 2. CRC Development

The risk to develop CRC is associated with age, genetic alterations, chronic disease history, e.g., inflammatory bowel disease, and lifestyle habits, including diet, alcohol consumption, smoking, lack of exercise, and alterations in the gut microbiome.

Primary CRC develops in the rectum and colon upon transformation of intestinal epithelial cells to a benign adenoma, followed by progression into carcinoma via accumulation of genetic and epigenetic abnormalities. During this progression, malignant cells actively modulate their microenvironment by interacting with immune cells, non-immune cells, such as vascular endothelium and stromal fibroblasts, and the extracellular matrix (ECM). In addition, the tumor can also affect distant locations by releasing extracellular vesicles, which favor metastatic dissemination by inducing the formation of premetastatic niches, particularly in the liver [2].

### 2.1. Genetic Background of CRC

CRC is a genetically heterogeneous disease with different subtypes characterized by specific molecular alterations, which play an important role in shaping the TME, tumor sensitivity to different treatments, and patients’ prognosis. The different structural abnormalities include chromosomal instability (CIN), microsatellite instability (MSI), and CpG island methylation, whereas mutations can target oncogenes, tumor suppressor genes, and genes related to DNA repair mechanisms [3]. Depending on the origin of these abnormalities, CRC can be classified as sporadic, inherited, or familial [4].

CIN is present in the majority of CRCs, is characterized by aneuploidy and loss of heterozygosity (LOH), and is associated with mutations in *KRAS*, *BRAF*, and *PIK3CA* and tumor suppressor genes, like *APC*, *SMAD4*, and *TP53* [5], which are often accompanied by aggressive clinicopathological characteristics [6]. MSI arises from defects in the DNA mismatch repair (MMR) system caused by mutations or epigenetic silencing of MMR genes, such as *MLH1*, *MSH2*, *MSH6*, *PMS1*, and *PMS2*. MSI is the hallmark of the Lynch syndrome, a hereditary form of CRC [7]. MSI-high (MSI-H) tumors exhibit high tumor mutational burden (TMB) and an increased number of tumor-infiltrating lymphocytes (TIL), particularly CD8+ cytotoxic T cells with an elevated expression of the programmed death receptor ligand 1 (PD-L1), which is associated with response to immune checkpoint (ICP) inhibitors (ICPi) [8]. The CpG Island Methylator Phenotype (CIMP) is characterized by hypermethylation of promoter’s CpG islands leading to gene silencing. CIMP is often associated with *BRAFV600E* mutations and occurs in right-sided CRC. Next to overlaps with MSI in sporadic CRC due to *MLH1* promoter methylation, CIMP tumors can be further subdivided into CIMP-low and CIMP-high CRC depending on the number of methylated promoters [9], but the clinical relevance of these subtypes is currently controversially discussed [10].

Next to alterations in these molecular markers, there exist several other mutations associated with the CRC phenotype. For example, mutations in the DNA polymerase epsilon (*POLE*) impair proofreading during DNA replication, giving rise to an ultra-mutated, but microsatellite-stable (MSS) phenotype. These tumors have strong immunogenic features, including a high frequency of TIL, increased PD-L1 expression, and response to ICPi therapy [11]. Loss-of-function mutations in *ARID1A*, a component of the SWI/SNF chromatin-remodeling complex, impair DNA repair and increase immunogenicity. Indeed, *ARID1A*-mutated CRCs demonstrate a higher TMB and enhanced immune cell infiltration, making them candidates for immunotherapy [12]. Mutations in the histone methyltransferases *KMT2C* and *KMT2D* present in CRC with an inflamed phenotype are associated with an epigenetic deregulation, altered immune responses, and improved patients’ outcomes upon treatment with ICPi [13]. Alterations in homologous recombination repair (HRR) genes, such as *BRCA1/2*, are found in a subset of CRCs. These tumors exhibit high genomic instability, elevated TMB, and an increased immune cell infiltration associated with an improved patient survival following ICPi treatment [14].

Using gene expression profiles instead of single mutations, CRC can be subdivided into four distinct groups, named the Consensus Molecular Subtypes (CMS), displaying different tissue architecture and responsiveness to various therapies [15]. CMS1 tumors are characterized by MSI-H, frequent *BRAFV600E* mutations, CIMP-high, and a strong immune activation, which is often associated with a poor response to conventional chemotherapy but sensitivity to ICPi. CMS2 tumors display CIN, activation of the WNT and MYC signaling pathways, and favorable responses to standard chemotherapies like FOLFOX. CMS3 tumors are defined by metabolic dysregulation and frequent *KRAS* mutations, but a low immune cell infiltration and an intermediate prognosis. These tumors may benefit from metabolism-targeted therapies. CMS4 tumors are characterized by stromal infiltration, activation of transforming growth factor-β (TGF-β) signaling, angiogenesis, and features of epithelial–mesenchymal transition (EMT), which are often accompanied by a worse prognosis, resistance to standard chemotherapy, but responsiveness to anti-TGF-β strategies.

### 2.2. Inflammation and CRC Cross-Talk with the Immune System

Inflammation is a critical physiological response that serves as a defense mechanism against pathogens and plays a significant role in tissue repair [16,17]. However, the nature of inflammation, whether acute or chronic, can have different implications for health, in particular in the context of CRC. In the acute phase, it is the body’s response to injury or infection and could effectively restore tissue homeostasis by generating locally active inflammatory mediator molecules. When inflammation persists, there is a transition into a chronic state, in which the sustained production of pro-inflammatory molecules and ongoing recruitment of inflammatory cells hampers the reestablishment of tissue homeostasis, leading to a deregulated inflammatory response. Chronic inflammation plays a pivotal role in the onset and progression of CRC. This is partially based on inflammation-related risk factors, such as obesity, lifestyle, tobacco use, alcohol consumption, and inflammatory bowel disease. It represents one of the hallmarks of cancer and promotes the acquisition of tumor cell characteristics, such as apoptosis suppression, uncontrolled growth, and immune evasion [18]. The increased production of pro-inflammatory agents like proteases, histamines, growth factors, chemokines, and cytokines creates a favorable environment for CRC development. Furthermore, anti-oxidant defense mechanisms are decreased, while the cellular vulnerability to oxidative stress is increased, leading to damage associated with reactive oxygen species. This prolonged state is a significant risk factor for CRC development, but also affects patients’ prognosis and response to therapy [19]. It causes alterations in the TME by changes in the dynamics of immune recognition and cytotoxicity [20]. For example, during tumor-related inflammatory processes, neoplastic and stromal cells recruit macrophages and neutrophils to the TME, thereby directing their polarization towards different phenotypes with immune-suppressive activity and pro-tumor functions [21,22]. High expression levels of inflammatory molecules are associated with the activation of the nuclear factor κ light chain enhancer of activated B cells (NFκB), which regulates the transcription of various inflammatory molecules, such as IL-1β, IL-6, COX2, and STAT3, leading to positive feedback of inflammation. STAT3 and NFκB activators play a crucial role in enhancing the production of pro-inflammatory mediators, including IL-6, from non-immune cells, such as fibroblasts and adipocytes. An intricate interplay between cell survival, migration, and inflammation underscores the significance of STAT3 and NFκB activation in orchestrating a cascade of events that contribute to inflammatory responses. This can be further amplified by hypoxia-induced cell death, which contributes to an inflammatory milieu, leading to progression and survival of CRC. Loss of p53 function affects epithelial integrity and activation of NFκB and STAT3 inflammatory pathways, while hypoxic stress and elevated TGF-β levels favor the differentiation of regulatory T cells (Treg), impairing antitumor immunity.

However, while inflammation can trigger carcinogenesis, it can also protect against cancer cells by regulating DNA damage repair and modulating the TME [23]. In this context, specific innate and adaptive immune cells, along with their effector molecules and pathways, can work together as tumor-suppressive factors [24].

### 2.3. The Role of the Gut Microbiota in CRC

The human gut is populated by a dense microbial community, including bacteria, viruses, fungi, and archaea, and it holds a rich repertoire of genetic diversity and metabolic function. This group of diverse microorganisms, termed the gut microbiota, exhibits significant individual variations in the composition and metabolic activity as a consequence of host genetics as well as environmental factors, such as diet, exercise, and hygiene. Despite being a dynamic system, the composition of the gut microbiota is relatively stable and has a high microbial diversity under healthy conditions, thereby maintaining a symbiotic relationship with the host, contributing to metabolic, immunologic, and protective function [25,26]. Several studies demonstrated that gut microbiota diversity is diminished under disease conditions, including CRC, and decreases with environmental factors promoting CRC risk, such as e.g., a Western-style diet (WSD; see below), urbanization, and smoking [27,28,29,30,31,32]. This reduction results in a perturbed compositional and functional configuration associated with disease states, termed dysbiosis. Dysbiotic patterns of the gut microbiome are commonly detected in fecal samples of CRC patients or individuals at high CRC risk, and are associated with tumor initiation, CRC development, and progression [29,33,34,35,36,37,38]. Notably, the transfer of fecal microbiota from CRC patients to germfree mouse models of CRC resulted in the promotion of colonic tumorigenesis and progression of intestinal adenomas [39,40]. Additionally, meta-analysis of fecal microbiome data revealed an increased abundance of several bacterial species in CRC patients [34,36,41], like pks+ *Escherichia coli*, *Enterococcus faecalis*, enterotoxic *Bacteroides fragilis*, and *Morganella morganii*. Furthermore, species from the oral microbiota, such as *Fusobacteria* and *Peptostreptococcus anaerobius*, are often detected in fecal samples of CRC patients, which may reflect the perturbed ecosystem in the gut providing niches for extraintestinal bacteria [42,43,44,45].

### 2.4. Link Between Nutrition and CRC

As the colon is in direct contact with the ingested food, nutrition has a significant impact on CRC carcinogenesis, progression, as well as on its responsiveness to treatment [46,47]. Additionally, there exists an interplay between diet and the gut microbiota that can further influence carcinogenesis by directly affecting epithelial cells and the immune system [48,49,50,51]. Indeed, microbiota dysbiosis, especially enhanced levels of *Fusobacterium nucleatum*, is linked to CRC carcinogenesis [52,53], whereas metastasis formation in the liver has been associated with different gut bacteria producing bile acids [54]. Consequently, by direct and indirect exposure, dietary choices can be pro- as well as anti-carcinogenic [55], thereby affecting prognosis and treatment efficacy in a supportive or inhibitory manner [56]. The typical Western diet is characterized by high saturated fat and carbohydrate content [57], not only resulting in obesity and metabolic syndrome [58], but also directly contributing to carcinogenesis due to the associated high levels of insulin and insulin like growth factor 1 as well as an increased oxidative stress [59,60], as demonstrated in mice fed with a high fat diet [61]. In contrast, the Mediterranean diet (MD) [62], comprising a high intake of vegetables, fruit, whole grains, fish, and unsaturated fatty acids [63], is associated with a reduced risk to develop a metabolic syndrome and CRC. Mechanistically, it has been demonstrated that MD not only changes the cell proliferation, but also affects epigenetic mechanisms [64], leading to the development of epigenetically active nutraceuticals, such as curcumin and various polyphenols from plants that influence the activity of histone deacetylases (HDAC) [65,66,67,68]. To assess the effect of nutrition, it is important to consider that the uptake of these compounds is rather scarce. Thus, many of the in vitro effects on cell cultures are likely not occurring under physiological conditions and might happen only under particular circumstances, such as, e.g., damaged epithelium [69].

Examples of food-derived factors that affect CRC development are short-chain fatty acids (SCFA), nutrient-derived peptides (NDP), and advanced glycation end products (AGE). SCFA are carboxylic acids with a chain length up to five that are either taken up with food or produced by gut microorganisms fermenting dietary fibers [70] and have protective effects against carcinogenesis [71]. Whereas most SCFA are rapidly absorbed by epithelial cells [72,73], residual amounts can enter the bloodstream and reach distant cells, such as phagocytes, B and T cells [74]. SCFA influence cellular metabolism by acting as substrates and modulators of mTOR [75] and interact with GPR41, GPR43, and GPR109A, thereby directly or indirectly influencing immune cells [76]. Furthermore, SCFA can exert epigenetic effects via the inhibition of HDAC [77], influencing, e.g., the Th polarization [78].

NDP can be released from nearly all nutritional protein sources under e.g., limited proteolysis occurring in the intestine. Next to their influence on the metabolism in distant tissues [79], including tumors and immune cells, NDP display multiple activities, such as anti-microbial, peptidase inhibitory, anti-oxidant, opioid, immunomodulatory, and anti-cancer [80]. The anti-cancer effects of NDP have been linked to their ability to induce apoptosis or disruption of the membrane of cancer cells due to their specific composition [81,82]. The immunomodulation is due to the capability of some NDP to act as angiotensin-converting enzyme (ACE) inhibitors and to modulate the expression of different angiotensin receptors (AGTR) on immune cells [83,84]. For instance, AGTR blockade contributes to an immunosuppressive TME [85], while angiotensin stimulation regulates the expansion and proliferation of antigen-specific CD8+ T cells [86,87] and the phagocytic phenotype of THP-1 cells [88]. Notably, AGRT are also expressed on cancer cells [89], with a differential regulation of AGRT 1 and 2 expression during the adenoma-adenocarcinoma sequence [90]. In contrast, AGRT blockade by Losartan inhibits CRC growth, which might be due to an induction of pro-inflammatory cytokines [91].

AGE are formed by the non-enzymatic modification of amino groups from proteins, lipids, and nucleic acids through the condensation with reactive aldehydes [92], the so-called Maillard reaction, followed by further oxidations and eliminations. AGE can be directly introduced through diet [93] or produced endogenously in conditions characterized by increased amounts of highly reactive, AGE-forming α-oxo-aldehydes, as often seen in cancers predominantly utilizing aerobic glycolysis [94]. In addition to cancer, AGE formation is associated with aging, diabetes, and cardiovascular diseases [95,96] and can inhibit enzyme and growth factor activity as well as crosslink matrix proteins. Both dietary and endogenous AGE can activate a multipattern receptor, named RAGE (receptor for AGE, also known as AGER), which is present on a multitude of cells, including immune cells, and frequently overexpressed on tumor cells. Since RAGE signaling can activate NFκB and induce, e.g., cytokine release, AGE can be considered pro-inflammatory factors [97]. Signaling through AGE can be regulated either by a soluble splice variant of RAGE (sRAGE) acting as a decoy receptor or by scavenger receptors mediating AGE removal [98,99]. Notably, glyoxalase I is highly expressed in CRC [100], and its abundance correlates with CRC development [101]. Modifying RAGE through ubiquitinylation using the natural compound Scutellarein inhibits carcinogenesis [102]. Furthermore, circulating AGE and sRAGE are associated with CRC mortality and dietary uptake of AGE-modified food peptides, thereby influencing cancer and its TME [103,104]. In sum, the relationship between nutrition and CRC is complex and multifaceted. Dietary choices can significantly influence the gut microbiota, which in turn affects CRC cancer development. Understanding of these connections can help in the development of dietary interventions to reduce CRC risks and improve treatment outcomes.

### 2.5. Microbiome Diet Interactions and CRC

Diet plays a major role in shaping the gut microbial community, with the WSD causing lower gut microbiota diversity [28,31,105,106] due to metabolic adaptation of microbes to WSD. Given the dynamic response of gut microbiota composition and metabolic function to the host nutrition, diet switch studies provided elegant proof-of-concept evidence for diet-mediated changes in microbial CRC risk patterns in healthy individuals [106], highlighting the potential to use dietary interventions for gut microbiota modulation in order to reduce CRC risk. For example, indigestible dietary fibers are fermented by specific members of the gut microbiota into SCFA, in particular butyrate, which shows anti-inflammatory and tumor-suppressive activity [107,108]. WSD is characterized by low fiber intake, which correlates with a higher CRC risk and is reflected by lower butyrate levels in feces of CRC patients and in high-risk cohorts [106,109,110,111]. Butyrate-producing bacteria cover a diverse group of bacteria, and lower abundances of butyrate-producing taxa were found in CRC patients and under high-risk conditions [35,106,111].

Furthermore, the high fat content of the WSD mediates an increased hepatic production of primary bile acids that, upon reaching the colonic lumen, are converted by specific gut bacteria to secondary bile acids, which show tumor-promoting activity. The predominant secondary bile acid deoxycholic acid (DCA) was shown to act as a tumor-promoting agent in rodent models of CRC [112,113] and is present in high levels in CRC patients and individuals at high CRC risk [31,36,110,114]. Consistently, bacteria being able to produce DCA by 7α-dehydroxylating activity are more abundant in fecal samples of CRC patients and healthy individuals at higher CRC risk due to the Western lifestyle [36,110].

Despite the evidence of an increased abundance of specific gut bacteria with pathogenic traits in fecal or tumor samples from CRC patients and the tumor-promoting effects of bacterial species in gnotobiotic models of CRC, a clear causal relationship between dysbiotic alterations of the gut microbiota and colorectal tumorigenesis remains a matter of debate. While there is convincing evidence that the gut microbiota of CRC patients is characterized by lower diversity, altered composition, and perturbed metabolic function when compared to healthy individuals, the investigation of causal effects in CRC pathogenesis requires studies that go beyond the comparison of diseased vs. healthy cohorts. This may be addressed by analyzing related changes in the gut microbiome of cohorts at different CRC risk (e.g., by longitudinal comparison of high- vs. low-risk cohorts) to unravel the causal role of the gut microbiota in CRC.

## 3. Cellular Metabolism and the Establishment of an Immunosuppressive TME

One of the hallmarks of cancer is the alteration of the cellular metabolism in order to cope with the enhanced anabolic demand of highly proliferating cells [115], with various changes taking place at the different phases of CRC tumorigenesis, namely the precancerous adenoma, the carcinomas, and the metastatic state [116]. Different oncogenes and tumor suppressor genes, such as *KRAS*, *p53*, *PTEN*, and components of the WNT pathway, are involved in such metabolic modulation. The mTOR pathway, which is activated in response to a variety of stress signals, e.g., hypoxia [117], the availability of nutrients and compounds like SCFA and curcumin [75,118,119], is involved in the regulation of metabolic pathways, such as glycolysis, the TCA cycle, and protein biosynthesis.

Like many solid tumors, CRC displays the Warburg effect, e.g., the usage of glycolysis for energy production even under oxygen-replete conditions. Genetic and pharmacological studies have demonstrated that the Warburg effect is not a simple bystander effect, but is required for tumor growth [116]. Indeed, it allows CRC cells to prioritize rapid ATP generation, biosynthesis of molecular precursors, and the maintenance of redox homeostasis, enabling a dynamic adaptation to microenvironmental stress.

Protein biosynthesis is also altered in cancer and associated with a deregulated expression of eukaryotic initiation factors (eIF) with tumor-specific expression patterns [120]. For example, differences in eIF expression are found between high- and low-grade CRC cases [121] with upregulation of eIF4E being an early event in CRC carcinogenesis [122]. Importantly, changes in eIF not only quantitatively, but also qualitatively regulate protein biosynthesis by affecting the ratio of cap- to IRES-mediated translation initiation of e.g., cMYC [123], which has a significant impact on the cellular metabolism [124,125]. Next to the stress-related protein translation, an altered preference for cap-initiated translation was demonstrated for class switching of B cells [126], which highlights the importance of the mTOR-eIF4EBP axis for the regulation of the immune system [127,128]. This is underscored by an association of T-cell activation with increased protein biosynthesis mediated by the transcription of eIF genes [129] and mTOR activity.

During CRC progression from the normal epithelium towards adenoma and carcinoma, alterations in lipid metabolism also occur [130], with some differences depending on the anatomical location of CRC, resulting in distinct prevalent species of sphingolipids between tumors developing in the colon and in the rectum [131].

In recent decades, it has been demonstrated that the altered metabolism also contributes to the remodeling of the TME to further promote tumor growth via inhibition of immune effector cells, recruitment of suppressive immune cells, and repolarization of immune and stromal cells toward pro-tumorigenic subpopulations [132]. For example, enhanced glycolysis of tumor cells results in a TME depleted of glucose, that is required by immune effector cells for antitumor activity, and enriched in lactate, that directly or via TME acidification impairs effector cells while promoting immune suppression mediated by myeloid-derived suppressor cells (MDSC) and Treg [133,134]. Recently, an additional role for lactate as a modulator of gene transcription upon conjugation with lysine residues of histones has been discovered [135]. Lactylation of histone H3K18 has been associated with an enhanced capability of CRC to metastasize to the liver via increased secretion of CXCL1 and CXCL5 [136] and conversion of macrophages into tumor-promoting cells by inhibition of the retinoic acid receptor expression and consequent secretion of IL6 [137]. In addition, non-histone proteins can undergo lactylation with consequences on their stability and/or functionality. Lactylation of RIG-I in macrophages inhibits the activation of NFκB, thus favoring their M2 polarization, a phenotype that could be reverted via 7-(carboxymethyl)-10-methyl-10H-phenothiazin-2-yl acetic acid [138].

In the MSS subtype of CRC, an asynchronous modification in the lipid metabolism due to MYC and SREBP2 leads to an accumulation of distal precursors of cholesterol. Released in the TME, they can bind to the RORγT transcription factor in naïve CD4+ T cells, promoting their polarization towards Th17 cells. This effect could be reduced in murine models by inhibition of Cyp51 [139]. Additionally, enhanced expression of sphingosine-1 phosphate not only in malignant cells, but also in endothelial cells and macrophages promotes angiogenesis and M2 polarization via the vascular endothelial growth factor (VEGF) and macrophage migration inhibitory factor, respectively [140].

Alterations in the amino acid (aa) metabolism further promote immune suppression in CRC [141]. For example, enhanced expression of indoleamine 2,3-dioxyglucose (IDO) inhibits T-cell function and infiltration due to depletion of tryptophan and production of kynurenine [142,143], whereas glutaminase can reduce the transcription of different components of the MHC class I antigen processing machinery and thus promote the escape from effector cells due to reduced antigen presentation [144]. Similarly, alterations in the nucleotide synthesis pathway, in particular enhanced levels of extracellular adenosine, are frequent in cancerous tissues and have inhibitory effects on multiple immune cells [145].

## 4. CRC Detection and Monitoring Using Liquid Biopsies (LB)

The term liquid biopsies (LB) was introduced over a decade ago by Pantel and co-authors to indicate the usage of whole blood instead of highly invasive solid tissue biopsies or non-specific blood stool tests to predict therapeutic outcomes as well as patients’ prognosis [146]. For CRC, LB offers a less invasive and faster processing time compared to the gold-standard colonoscopy paired with solid tissue biopsy retrieval. Thus, the compliance of patients to preventive screening as well as therapeutic monitoring is much higher using LB [147,148]. However, the ideal scenario involves leveraging blood tests to increase screening participation, while simultaneously promoting stool and endoscopy screening [149].

In recent years, several in vitro diagnostic (IVD)-certified LB tests have been developed for CRC screening prior to colonoscopy as well as for therapeutic monitoring via detection of circulating tumor cells (CTC) or circulating tumor-derived DNA (ctDNA) [150,151] (Table 1). CTC are released into the bloodstream by primary and metastatic tumors and can be identified by flow cytometry as CD45-negative (e.g., non-immune) cells expressing tumor-associated markers, such as the epithelial cell adhesion molecule EpCAM and cytokeratins. A threshold of three CTC per 7.5 mL whole blood can predict the clinical outcomes in patients with metastatic CRC [150]. Using semi-quantitative qPCR on blood, the presence of some CTC-associated markers, such as plastin-3, can differentiate CRC samples from benign inflammatory intestinal disease [152].

ctDNA enters the bloodstream through cellular apoptosis or necrosis [159]. In comparison to cell-free DNA released from healthy blood cells, ctDNA is present at very low amounts [160], requiring highly sensitive analytical methods, such as quantitative or digital real-time PCR or deep next-generation sequencing (NGS) to identify tumor-specific mutations. Depending on the IVD assay employed, ctDNA detection can be implemented for early screening, guiding therapy selection, or monitoring of minimal residual disease (MRD) during and after treatment (Table 1).

LB are also implemented to monitor the cellular components in peripheral blood as well as inflammatory markers of CRC patients [161]. Through multicolor flow cytometry and/or multiplex ELISA, the composition and function of cellular components, along with the profile of cytokines/chemokines, can serve as prognostic markers for predicting OS, but also recurrence, as well as therapy response [162,163].

Some of the alterations in tumor metabolism also have systemic consequences on the levels of specific metabolites, such as different aa [164] and cholesterol precursors [139]. These can be detected in LB to distinguish patients from healthy donors [165] and to identify different disease stages, such as polyps versus carcinoma [166]. Additionally, they can be used for patients’ monitoring prior to, during, or after therapy to identify treatment resistance. Ongoing development of additional diagnostic tests and additional units in technology may further enhance the role of LB in oncology.

## 5. Current Strategies for Treatment of CRC

Over recent decades, the treatment of patients with metastatic CRC has become increasingly interdisciplinary with a number of novel systemic treatments. Next to the complete surgery, radiotherapy, chemotherapy, targeted treatments, and more recently, different forms of immunotherapy are implemented in CRC patients. Despite the rapid development of biomarker-driven treatment options for this disease, in particular for specific molecular CRC subtypes, challenges persist in achieving optimal patients’ outcomes and complete resection, including the locoregional lymphatic drainage system, remains crucial for achieving a cure.

### 5.1. Surgery

Colon cancer: Endoscopically removed malignant polyps may be managed with surveillance when favorable histopathologic features are present, including low-grade differentiation, absence of angiolymphatic invasion, and negative resection margins [167]. For locally resectable, non-metastatic colon carcinomas, primary segmental colectomy with en bloc removal of the regional lymphatic drainage basin remains the standard of care [167,168]. This applies to both obstructing and non-obstructing tumors. Although evidence is not entirely consistent, several studies indicate that patients presenting with obstruction may benefit from treatment by specialized colorectal surgeons, with fewer stomas, fewer anastomotic leaks, and improved long-term outcomes. To facilitate specialized surgical management, an obstructive ileus may initially be addressed with the creation of a stoma, allowing for planned elective resection [169]. As an alternative bridging strategy, endoscopic stent placement has also been shown to be effective [169]. Despite increasing efforts to perform primary anastomoses in emergency sigmoid or rectal cancer surgery, the Hartmann procedure continues to play an important role in critically ill patients at high risk of anastomotic leak [169]. For node-positive disease or certain high-risk constellations, adjuvant systemic therapy is recommended. Locally unresectable tumors should undergo neoadjuvant systemic therapy followed by resection when a sufficient response is achieved. Patients with clinical T4b disease or distant non-locoregional lymph node metastases should likewise receive neoadjuvant therapy prior to surgery [167,168]. During resection of the primary tumor, involved adjacent organs may be removed en bloc when necessary.

Rectal cancer: Early, small, and clinically mobile rectal carcinomas (T0–1, N0) may be treated with endoscopic or local surgical excision [170]. However, tumors with submucosal invasion greater than 1000 µm present an estimated 12% risk of lymph node metastasis and are therefore not ideal candidates for local excision [170,171]. International guidelines establish histopathologic criteria required to define a curative local resection; if these criteria are not met, radical resection is indicated [172]. All other rectal cancers located in the upper, middle, or lower third of the rectum are treated with transabdominal resection and en bloc removal of regional lymph nodes. Lymphadenectomy is performed through total mesorectal excision, which is essential for reducing local recurrence [170]. A diverting stoma is frequently created to limit the need for reoperation in the case of anastomotic leakage. For tumors of the upper rectum, partial mesorectal excision with a 5-cm distal margin is sufficient [172]. Anastomotic leakage occurs in approximately 8.7% of cases even in specialized centers [173]. Many of these complications can be managed successfully through endoscopic measures, including vacuum therapy. Preoperative chemoradiation may result in complete clinical response rates of up to 27% when total neoadjuvant therapy (TNT) is employed. This enables selected patients to undergo non-operative management, with surgery reserved for regrowth [174]. However, since TNT was originally studied in locally advanced disease, recent recommendations extending its use to T1–2, N+ tumors should be critically evaluated and further validated [171]. 

Current innovations and future trends in colorectal surgery include: • broader adoption of laparoscopic and robotic surgery as preferred operative approaches, • improved adherence to evidence-based guidelines, • routine discussion of cases in multidisciplinary tumor boards, • expansion of Enhanced Recovery After Surgery pathways and prehabilitation programs, • implementation of image-guided and emerging AI-assisted surgical techniques, and • development of remote robotic surgery capabilities.

### 5.2. Chemo- and Radiotherapy in CRC

Chemotherapy with oxaliplatin- or irinotecan-based therapies is still the gold standard to increase OS in patients with metastatic CRC, who cannot have surgery [175,176,177,178,179]. Patients with locally advanced CRC receive neoadjuvant chemotherapy before surgery [180], but only 15% of them achieve complete pathological remission [181], with better responses obtained in high-risk stage II and III CRC patients [182]. In addition to the severe side effects associated with the high systemic toxicity of chemotherapy, an additional challenge is the presence of intrinsic or acquired resistance to the treatment [183]. To improve the therapeutic effect of cytotoxic treatments, novel delivery strategies were investigated, including nanoparticle-based drug carriers and co-formulated immunomodulatory compounds designed to enhance tumor-selective drug accumulation while at the same time limiting systemic toxicity. Such approaches can allow simultaneous modulation of tumor cells and immune responses [184,185,186].

For instance, nanoemulsion-based delivery methods have been shown to increase the solubility, stability, and systemic application of hydrophobic medicines, and to enable co-delivery of immunostimulatory agents. These methods have been found to increase immunogenic cell death and antitumor immunity. Similarly, sophisticated nanocarrier platforms such as liposomal and polymer-based systems provide regulated release of drugs and better tumor targeting, which could result in increased therapeutic efficacy and less off-target effects [187,188].

Radiotherapy (RT) can induce immunogenic cell death, which is accompanied by the release of DAMPs, increased antigen presentation, and priming of T cells [189]. Preclinical data demonstrated that radiation potentially can increase the susceptibility of tumor cells to T-cell-mediated elimination. On the other hand, it could exert negative feedback mechanisms, such as upregulation of ICP [190].

It is noteworthy that radiation has a limited efficacy in treating disseminated metastatic CRC, primarily due to the complex interactions with the TME and the immune responses. Nonetheless, selective internal radiation therapy has demonstrated potential benefit, such as downsizing and downstaging metastatic CRC [191,192]. Furthermore, stereotactic body radiation has been successfully implemented for the management of oligometastatic patients, especially those with liver metastasis, and can provide local tumor control without significant (systemic) toxicity [193].

### 5.3. Targeted Therapy

In clinical practice, currently actionable biomarkers guiding targeted therapy include components of the epidermal growth factor receptor (EGF-R) signaling pathway, like EGF-R, BRAF, and RAS [194], MSI-H/dMMR status, and rare NTRK fusions. In contrast, several additional molecular pathways discussed in this review, including metabolic reprogramming, inflammatory signaling, and microbiome-mediated mechanisms, remain largely investigational and are currently evaluated mainly in translational or early-phase clinical studies [195,196].

In addition, an amplification and overexpression of the human epidermal growth factor receptor 2 (*HER2*) has been identified in approx 6% of metastatic CRC patients with wild-type *RAS* CRC [197], which provides the rational for HER2-targeted regimens for these patients [198]. Furthermore, the VEGF pathway, which is frequently upregulated in CRC, has been targeted by multiple drugs targeting VEGF or angioprotein-2 [194,199,200]. Interestingly, the use of anti-EGF-R and anti-VEGF treatments is dependent on the presence of mutations in the epigenetic regulator *ARID1A* and is used as a predictive biomarker [201]. Currently, innovative and more specific tyrosine kinase inhibitors are implemented in CRC patients [202]. Despite targeted therapies often prolong progression-free survival (PFS) of metastatic CRC patients, the development of drug resistance remains a significant barrier to long-term benefits [203].

### 5.4. Immunotherapy of CRC

Over the last few decades, cancer immunotherapy has revolutionized patient treatment with the induction of durable responses, but with differences among tumor histotypes. Indeed, the success of immunotherapy highly depends on the structure and composition of the individual tumor, with the extent, type, and spatial distribution of the immune cell infiltrate as well as the TMB playing an important role, as demonstrated for melanoma, lung, and bladder cancer patients [204].

In line with these data, and as summarized in Table 2, responses to ICPi, such as pembrolizumab, nivolumab, ipilimumab, and dostarlimab, are partially notable in the MSI-H or MMR deficient (dMMR) subgroup of both metastatic and early disease. In contrast, MSS and MMR proficient (pMMR) CRC, which represent the majority of cases, are mostly unresponsive to ICPi monotherapy, with exceptions for tumors harboring mutations leading to a TMB high phenotype [11].

This poor response can be mainly ascribed to the low neoantigen burden of MSS tumors, reduced T-cell infiltration, defective antigen presentation, the presence of immunosuppressive myeloid populations within the tumor microenvironment and/or an impaired capability of immune cells to target tumor cells. In addition, stromal exclusion and immunosuppressive cytokine signaling are also involved in the “cold” immunological phenotype frequently observed in MSS CRC [195,205,206].

Furthermore, resistance to ICPi has also been linked with the presence of liver metastasis, as shown in the IOLite trial [207,208].

**Table 2:** Summary of currently available immunotherapy trials in CRC.

**A. dMMR/MSI-H CRC (ICPi sensitive)**				
**Study/Trial**	**Population/Stage**	**Therapy**	**Key Findings**	**Ref.**
KEYNOTE-164 (Phase II)	Metastatic dMMR/MSI-H CRC, ≥1 prior lines	Pembrolizumab	ORR ^§^ ~33% in both cohorts; CR in 3 and 8 patients; median OS: 31.4 mo (Cohort A), not reached (Cohort B); 3-year OS: 49–52%; Grade 3–4 AE: 13–16%	[209]
KEYNOTE-177 (Phase III)	First-line metastatic dMMR/MSI-H CRC	Pembrolizumab vs. chemotherapy	Median PFS: 16.5 vs. 8.2 mo (HR: 0.60); fewer grade ≥ 3 AE (22% vs. 66%); improved QoL	[210,211]
KEYNOTE-016 (10y)	dMMR solid tumors, including CRC	Pembrolizumab	ORR: 58%; median PFS: 34.9 mo; median OS: 80.8 mo; 10-year OS: 47.4%	[212]
CheckMate-142 (Phase II)	Metastatic dMMR/MSI-H CRC	Nivolumab ± Ipilimumab	Nivolumab: ORR ~34%, median PFS ~6.6 mo; with ipilimumab: ORR 58–65%, DCR ≥ 78%; 5-year PFS/OS > 70%	[213]
NICHE-2 (Phase II)	Stage II/III dMMR colon cancer (neoadjuvant)	1 × Ipilimumab + 2 × Nivolumab	98% pathologic response; 68% CR; 3-year DFS ~100%; low grade 3–4 toxicity	[214]
Dostarlimab Trial	Locally advanced dMMR/MSI-H rectal cancer	Dostarlimab (neoadjuvant)	100% clinical complete response; no grade ≥ 3 AE; all patients avoided surgery and chemoradiation	[215])
**B. MSS/pMMR CRC (ICPi refractory)**				
**Study/Trial**	**Population/Stage**	**Therapy**	**Key Findings**	**Ref.**
KEYNOTE-016/CheckMate-142 (MSS cohorts)	MSS/pMMR CRC	anti-PD-1/anti-CTLA-4	ORR near 0%; confirms lack of ICPi monotherapy efficacy	[216,217,218]
IOLite (Phase II)	Refractory MSS CRC ± liver metastases	anti-PD-1 + anti-LAG-3	Benefit mainly in patients without liver metastases; hepatic involvement linked to systemic ICPi resistance	[207]
Preclinical/translational strategies	MSS CRC (cold tumors)	ICPi + TME-modulating agents	Combinations with regorafenib, oncolytic viruses, or cytokines may enhance immune infiltration and overcome resistance	Conceptual/preclinical (no specific trial cited)

^§^ AE, adverse events; CR, complete response; DFS, disease free survival; PFS, progression free survival; ORR, objective response rate.

Preclinical and translational data support the rationale for combining ICPi with agents that inhibit oncogenic pathways and modulate the TME, such as anti-angiogenic therapies, oncolytic viruses, or targeted cytokine delivery. These approaches aim to reverse tumor intrinsic immune escape mechanisms, enhance immune cell infiltration, and overcome the “cold” tumor phenotype typically seen in MSS CRC. For example, the addition of regorafenib, a multi-kinase inhibitor, to the blockade of the programmed death receptor 1 (PD-1) is being actively investigated, as it may normalize tumor vasculature and promote T-cell infiltration [219]. In the phase II Atezo TRIBE Trial, a monoclonal antibody (mAb) directed against PD-L1 was combined with chemotherapy and targeted therapy [220].

In addition to ICPi, CRC is currently treated with active cancer vaccines. A number of antigenic stimulants are investigated in multiple clinical trials. For example, a vaccination trial consisting of an off-the-shelf peptide cocktail of six synthetic peptides with 12 unique epitopes derived from seven conserved cancer testis antigens has been implemented (NCT 03391232) and the immunogenicity and efficacy monitored. Four out of 11 patients reached an objective response and/or a durable clinical benefit with the induction of a long-lasting CRC-specific T-cell response and enhanced infiltration of CD8+ cytotoxic T cells. Furthermore, a vaccine based on replication-deficient human type recombinant adenovirus as a vector induced a specific immune response against the encoded guanyl cyclase. The safety and efficacy of this approach were evaluated in a phase I clinical trial (NCT 01972737) with ten patients. The autologous vaccine oncoVAX, implementing the patient’s own tumor cells as vaccine in order to provide all relevant tumor-associated antigens to the immune system, has been provided after surgery in a phase III clinical trial to determine the current status of CRC following surgery (NCT 02448173).

Concerning the adoptive cell therapy, encouraging preliminary results have been obtained both with the transfer of TIL and T cells engineered either with chimeric antigen receptors (CAR) or with tumor-specific T-cell receptors. In the CAR T-cell therapy setting, constructs targeting the carcinoembryonic antigen are being implemented. Furthermore, the safety as well as efficacy of CAR T cells further engineered to express PD-L1 or NKG2D receptor is being investigated. In the setting of TIL transfer, novel encouraging results were obtained upon the identification within a patient’s TIL of polyclonal CD8+ T-cell responses against the G12D mutation of *KRAS*. Clinical transfer of such TIL into the patient resulted in regression of multiple metastases [221]. Despite encouraging results from these emerging approaches, several barriers limit their broader clinical translation. These include tumor heterogeneity, limited predictive biomarkers, immune-suppressive tumor microenvironments, manufacturing complexity of cellular therapies, and the need for improved patient selection strategies. Therefore, further translational studies and biomarker-driven clinical trials are essential to identify patients who may benefit from these innovative treatments [195,222,223].

## 6. New Approaches for (Metastatic) CRC

The landscape of metastatic CRC treatment is evolving, enabling the interference of functional niches and correlations between metabolic markers. In light of the heterogeneity of CRC and the existence of intrinsic as well as acquired resistance to the different “standard” therapeutic treatments, it is crucial (i) to improve patients’ stratification to the most effective therapeutic approach by identifying predictive biomarkers and (ii) to optimize the treatment modalities, e.g., combinatorial treatments or treatment sequences. In the following sections, we will discuss novel biomarkers and therapeutic interventions targeting the cellular metabolism or the host nutrition.

### 6.1. Cellular Metabolism as Biomarker and/or Therapeutic Target

Recent advances in sequencing technologies, including bulk and single-cell sequencing as well as spatial transcriptomics, which retain spatial information of the investigated cells, have allowed a comprehensive analysis of tumor tissues. Correlation of such sequencing data with clinical parameters, including therapy outcomes, allows the determination of markers for patients’ stratification to therapy and, upon understanding the molecular/immunological mechanisms underlying this correlation, to identify new therapeutic targets.

Evaluation of CRC sequencing datasets identified different prognostic markers/signatures belonging to multiple metabolic pathways, most of which also correlated with the cellular composition of the tumor ecosystem. Examples are signatures associated with lactate [224,225], general lipid metabolism [226,227], in particular with the fatty acid (FA) metabolism [228,229], aa metabolism in general [230,231,232], or specifically related to glutamine and glutaminase expression [233,234] as well as purine metabolism [235]. Predictive markers have also been found in datasets with treatment information. For example, lactate signatures correlated with responses to anti-PD-L1 [236], different aa signatures were found in responders to anti-PD-1, anti-CTLA4, or anti-PD-L1 therapies [237,238,239,240], and changes in nucleotide metabolism predicted response to anti-PD-1 [241]. Implementation of spatial transcriptomics allowed to associate those signatures with specific cellular components of the TME. For example, the enhanced aa metabolism within the epithelial cells correlated with a worse response to anti-PD-1 in CRC patients [238], whereas in a mouse model, tumor-specific enhanced expression of genes involved in cholesterol synthesis was responsible for the unresponsiveness to anti-PD-1 due to CD8+ T-cell exclusion [242].

The cellular metabolism is also influencing the response and/or resistance to chemotherapy. Enhanced lactylation correlates with resistance to chemotherapy (reviewed in [243,244]). Mechanistically, resistance to 5-fluoro uracil (5′-FU) is due to the enhanced stability of lactylated CEACAM6 [245], whereas bevacizumab resistance correlated with enhanced autophagy in response to lactylation of histone H3K18 [246]. A signature related to glutamine metabolism can predict response to different chemotherapeutics [240], while enhanced IDO expression causes resistance to cetuximab [247] and 5′-FU [248]. The purine metabolism and, in particular, higher levels of guanosine monophosphate, caused resistance to oxaliplatin, and can be reversed by targeted inhibition of inosine 5’-monophosphate dehydrogenase type II [249].

Recent advances in machine learning algorithms have enabled the prediction of treatment responsiveness based on gene expression profiles without direct therapy information. For example, clustering patients from the TCGA dataset based on glucose metabolism genes has highlighted distinct genes involved in the responsiveness to multiple targeted as well as immuno-therapies [250]. A lactylation-related gene signature applied to different datasets suggested enhanced responsiveness to anti-PD-1 mAb for the low group and differential sensitivity to various chemotherapy agents between the high and the low group [251]. Reduced possible responsiveness to PD-1 or CTLA4 blockade, as well as to different chemotherapy approaches, was also inferred based on a nucleotide-metabolism-related gene signature [252], with two genes of the signature that were identified as possible therapeutic targets for the high-risk group [252].

In line with the prognostic/predictive role of different metabolic enzyme/metabolite/pathways, different therapeutic strategies targeting the abnormal metabolic pathways are currently under investigation in preclinical models as well as in clinical trials [253]. Tumor cells develop multiple adaptations in response to therapy-induced stress. In order to mitigate treatment resistance to radiotherapy and chemotherapy, therapeutic strategies for the metabolic reprogramming of CRC have been proposed [254] with a particular focus on the Warburg effect [255]. The anti-diabetic drug metformin has been associated with a reduced risk of CRC and has therefore been proposed as a therapeutic agent for this disease, alone or in combination with ICPi [256]. Mechanistically, different in vitro as well as preclinical mouse models have suggested that the metformin effect might be due to the inhibition of glycolysis and mitochondrial function [257], a reprogramming of tryptophan metabolism leading to enhanced CD8+ T-cell effector functions [258], and/or changes in the mevalonate pathway leading to reduced M2 polarization and recruitment of MDSC [259]. Additionally, a direct role of metformin on the composition of the gut microbiome with an enhanced amount of SCFA-producing species was demonstrated in murine models [260]. Many attempts are currently performed to target the enhanced lactate production [261] and reverse the acidification of the TME [262], whereas other strategies take advantage of it. For example, since the activity of the ICP VISTA at low pH is altered [263], a new pH-selective blocking mAb has been isolated and is currently clinically tested [264].

Inhibition of glutaminase in CRC-bearing mice enhanced the production of reactive oxygen species [144], reduced MDSC recruitment [265], and inhibited stromal activation and angiogenesis [266], leading to clinical trials combining the CB-893 glutaminase inhibitor with a MAPK inhibitor or with 5’-FU [267]. Inhibition of the altered lipid metabolism using a FASN inhibitor has been shown to suppress CRC cell growth, induce apoptosis, and reduce liver metastasis [268].

Furthermore, many attempts are also focusing on targeting the dysregulated metabolism in the TME, such as reverting the M2 polarization associated with CRC either via natural compounds favoring expression of nitrogen oxygen species over arginase [269] or combining IFN-γ and the mevalonate pathway inhibitor zoledronate [270].

However, it should also be noted that there exist several factors limiting the efficacy of these treatments, while targeting cancer metabolism holds promise. Due to the high toxicity of this approach, a number of studies were premature and were terminated [271]. In addition, the inter- and intra-tumoral heterogeneity can significantly dampen these treatments.

### 6.2. Nutritional Markers and Intervention

Many different studies have correlated the microbial content of stool probes from CRC patients with outcome to ICPi (reviewed in [272]) as well as chemo- or targeted-therapy (reviewed in [273]), revealing significant associations. For example, *Fusobacterium nucleatum* has been linked with a worse patient outcome due to an enhanced level of succinic acid, impairing immune response and sensitivity to immunotherapy [274], whereas *Bacteroides fragilis* promotes the synergistic effect between radiotherapy and immunotherapy [275]. As a consequence, different therapeutic approaches, such as fecal transfer, provision of probiotic or nutritional interventions, have been proposed in order to “re-normalize” the microbiome and consequently also the immune system of CRC patients.

Fasting-mimicking diet (FMD) has been demonstrated to boost antitumor immunity via modulation of the intestinal microbiota as well as tumor metabolic rewiring due to reduced nutrient availability in the TME. In murine models, FMD has been shown to improve survival by favoring colonization by *Lactobacillus johnsonii* [276] or *Bifidobacterium pseudolongum* [277], with the latter producing enhanced level of L-arginine, which promote the development of protective tissue-resident CD8+ T cells [277]. Additionally, FMD reduced the switching of B cells toward IgA-producing cells, which were otherwise suppressing CD8+ T cells [278]. In vitro studies demonstrated that FMD can induce tumor cells to enter a quiescent, slow cycling state, which can develop resistance to chemotherapy, but that is more sensitive to ferroptosis, thereby promoting combination therapy [279]. Bacteria are also being manipulated to improve their properties, for example, by coupling to their surface via a reactive oxygen species-responsive linker optical reactive nanoparticle loaded with an adjuvant [280].

Focusing only on diet interventions, various combinations of ω-unsaturated FA, aa (arginine, glutamine), and anti-oxidants are proposed to stimulate the immune response prior to surgery [281]. Such immunonutritional interventions have a positive effect on post-surgery complications, length of hospital stays, and humoral T-cell responses [282], inducing also significant changes in the TME, such as higher numbers of CD8+ cytotoxic T cells, Th cells, NK cells, as well as professional antigen-presenting cells [283].

Administration of specific metabolites, such as β-hydroxybutyrate, has been suggested as an option for prevention and/or treatment of CRC [284,285]. In addition, curcumin, a phenolic compound from curcuma with anti-carcinogenic [286], anti-inflammatory [287,288], and cholesterol-lowering activity [289], has been combined with radiotherapy, resulting in increased abscopal effects on non-irradiated tumors and improved efficacy due to enhanced immune responses [290]. In order to improve the otherwise low uptake of curcumin, nanoparticles or liposomes have been developed [291,292,293].

## 7. Conclusions

Current challenges in CRC therapy are tumor heterogeneity, treatment resistance, the limited number of biomarkers for proper patient stratification, adverse effects of therapies, and limited access to novel therapies due to the high costs and limited health infrastructure.

It is generally accepted that one hallmark of CRC is an altered metabolism associated with an increased proliferative capacity as well as significant changes in the TME. CRC adapt their metabolism during disease initiation and progression. This is primarily driven by alterations in canonical oncogenes and tumor suppressor genes, which enhance the energy requirement of CRC, thereby acquiring a selective advantage. Due to these data, targeting the metabolism in CRC might provide future approaches to improve the patients’ outcome after identifying metabolic vulnerabilities in the TME. This will lead to the design of tailored therapies on the basis of genetically defined metabolic targets, thereby leading to metabolic reprogramming during cancer progression in nutrient-limited environments. Notably, novel clinical targets, such as the phosphocreatine transporter SLC68A, are already in clinical trials. Furthermore, other metabolic adaptations of CRC are prime targets for chemoprevention and treatment with a potential for combination therapies. However, there exist various challenges to enhance the efficacy of metabolic drugs due to the metabolic heterogeneity and adverse effects, high plasticity, and the complex tumor/TME interactions. In addition, a novel class of therapies arises from the rapidly increasing microbiome field, which may also allow disease prevention and management. Nonetheless, there is an urgent need for preclinical and clinical research in this field in order to prove that metabolic adaptations are indeed the main drivers of tumor initiation and progression.

Recent advances in nanoemulsion-based and nanoparticle-driven co-delivery systems highlight their potential to improve drug solubility, reduce systemic toxicity, and enhance antitumor immune responses. These approaches may facilitate the clinical translation of combination therapies [187,188].

Future therapeutic strategies will therefore likely combine molecularly stratified therapies with advanced delivery systems and biomarker-guided patient selection to improve treatment efficacy while minimizing systemic toxicity [294,295].

## Figures and Tables

**Table 1:** Overview of IVD-certified molecular biological and NGS-based assays for CRC monitoring.

Assay Model	Manufacturer	Biomaterial	Diagnostic Quality Attributes	Approved for	Ref.
CELLSEARCH^®^ Circulating Tumor Cell Kit	Menarini Silicon Biosystems (Huntingdon Valley, PA, USA)	CTC	>3 CTC/7.5 mL whole blood at baseline and follow-up predict reduced PFS and OS; sensitivity 27%, specificity 93%, positive predictive value 53%, negative predictive value 81%		[150]
ColoScape™ Colorectal Cancer Detection Test	Diacarta (Hayward, CA, USA)	ctDNA, qPCR for 19 mutations (*APC*, *KRAS*, *BRAF*, and *CRNNB1*)	Sensitivity 92.2%, specificity 100%	Screening for early CRC	[153]
Plasma-SeqSensei™ Solid Cancer IVD Kit	Sysmex (Kobe, Japan)	ctDNA, NGS for *BRAF*, *EGF-R*, *KRAS*, *NRAS*, and *PIK3CA*	sensitivity 0.07% for mutated allele fractions	CRC disease monitoring, e.g., detecting MRD	[154]
FoundationOne^®^ Liquid CDx assay	Roche (Rotkreuz, Switzerland)	ctDNA, NGS for 324 genes	limit of detection of 0.40% variant allele fraction, sensitivity 99%		[155]
Guardant^®^-Shield screening kit	Guardant health (Palo Alto, CA, USA)	ctDNA, NGS	sensitivity 83.1–87.5%, specificity 90%	CRC screening prior to colonoscopy	[156]
Guardant^®^ 360 kit	Guardant health (Palo Alto, CA, USA)	ctDNA, NGS of 73 genes	n/d	CRC targeted-therapy decision	[157]
Guardant^®^ Reveal kit	Guardant health (Palo Alto, CA, USA)	ctDNA, NGS	sensitivity 81%, specificity 98.2%	MRD monitoring	[158]

## Data Availability

No new data were created or analyzed in this study. Data sharing is not applicable to this article.

## Abbreviations

The following abbreviations are used in this manuscript:

5′-FU	5-fluoro uracil
aa	amino acid
ACE	angiotensin-converting enzyme
AE	adverse events
AGE	advanced glycation end products
AGTR	angiotensin receptors
CAR	chimeric antigen receptors
CIMP	CpG Island Methylator Phenotype
CIN	chromosomal instability
CMS	consensus molecular subtypes
CR	complete responses
CRC	colorectal cancer
ctDNA	circulating tumor-derived DNA
CTC	circulating tumor cells
DCA	deoxycholic acid
DFS	disease-free survival
dMMR	MMR deficient
ECM	extracellular matrix
EGF-R	epidermal growth factor receptor
eIF	eukaryotic initiation factors
EMT	epithelial–mesenchymal transition
FA	fatty acid
FMD	fasting-mimicking diet
HDAC	histone deacetylases
HER2	human epidermal growth factor receptor 2
HRR	homologous recombination repair
ICP	immune checkpoint
ICPi	immune checkpoint inhibitor
IDO	indoleamine 2,3-dioxyglucose
IVD	in vitro diagnostic
LB	liquid biopsies
LOH	loss of heterozygosity
mAb	monoclonal antibodies
MD	Mediterranean diet
MDSC	myeloid-derived suppressor cells
MMR	mismatch repair
MRD	minimal residual disease
MSI	microsatellite instability
MSI-H	MSI-high
MSS	microsatellite-stable
NDP	nutrient-derived peptides
NFκB	nuclear factor κ light chain enhancer of activated B cells
NGS	next-generation sequencing
ORR	objective response rate
OS	overall survival
PD-1	programmed death receptor 1
PD-L1	programmed death receptor ligand 1
PFS	progression-free survival
pMMR	MMR proficient
POLE	polymerase epsilon
RAGE	receptor for AGE
RT	radiotherapy
SCFA	short-chain fatty acids
sRAGE	soluble RAGE
TGF-β	transforming growth factor β
TIL	tumor-infiltrating lymphocytes
TMB	tumor mutational burden
TME	tumor microenvironment
TNT	total neoadjuvant therapy
Treg	regulatory T cells
VEGF	vascular endothelial growth factor
WSD	Western-style diet

## References

[1] Matsuda T., Fujimoto A., Igarashi Y. (2025). Colorectal Cancer: Epidemiology, Risk Factors, and Public Health Strategies. Digestion.

[2] Li Y., Wang H., Mao D., Che X., Chen Y., Liu Y. (2025). Understanding pre-metastatic niche formation: Implications for colorectal cancer liver metastasis. J. Transl. Med..

[3] Fearon E.R., Vogelstein B. (1990). A genetic model for colorectal tumorigenesis. Cell.

[4] Lynch H.T., de la Chapelle A. (2003). Hereditary colorectal cancer. N. Engl. J. Med..

[5] Markowitz S.D., Bertagnolli M.M. (2009). Molecular origins of cancer: Molecular basis of colorectal cancer. N. Engl. J. Med..

[6] Jang S., Hong M., Shin M.K., Kim B.C., Shin H.-S., Yu E., Hong S.-M., Kim J., Chun S.-M., Kim T.-I. (2017). KRAS and PIK3CA mutations in colorectal adenocarcinomas correlate with aggressive histological features and behavior. Hum. Pathol..

[7] Gómez-Molina R., Martínez R., Suárez M., Peña-Cabia A., Calderón M.C., Mateo J. (2025). Lynch syndrome and colorectal cancer: A review of current perspectives in molecular genetics and clinical strategies. Oncol. Res. Featur. Preclin. Clin. Cancer Ther..

[8] Le D.T., Uram J.N., Wang H., Bartlett B.R., Kemberling H., Eyring A.D., Skora A.D., Luber B.S., Azad N.S., Laheru D. (2015). PD-1 Blockade in Tumors with Mismatch-Repair Deficiency. N. Engl. J. Med..

[9] Ogino S., Kawasaki T., Kirkner G.J., Ohnishi M., Fuchs C.S. (2007). 18q loss of heterozygosity in microsatellite stable colorectal cancer is correlated with CpG island methylator phenotype-negative (CIMP-0) and inversely with CIMP-low and CIMP-high. BMC Cancer.

[10] Duta-Ion S.G., Juganaru I.R., Hotinceanu I.A., Dan A., Burtavel L.M., Coman M.C., Focsa I.O., Zaruha A.G., Codreanu P.C., Bohiltea L.C. (2024). Redefining Therapeutic Approaches in Colorectal Cancer: Targeting Molecular Pathways and Overcoming Resistance. Int. J. Mol. Sci..

[11] Domingo E., Freeman-Mills L., Rayner E., Glaire M., Briggs S., Vermeulen L., Fessler E., Medema J.P., Boot A., Morreau H. (2016). Somatic POLE proofreading domain mutation, immune response, and prognosis in colorectal cancer: A retrospective, pooled biomarker study. Lancet Gastroenterol. Hepatol..

[12] Tokunaga R., Xiu J., Goldberg R.M., Philip P.A., Seeber A., Battaglin F., Arai H., Lo J.H., Naseem M., Puccini A. (2020). The impact of ARID1A mutation on molecular characteristics in colorectal cancer. Eur. J. Cancer.

[13] Liu R., Niu Y., Liu C., Zhang X., Zhang J., Shi M., Zou W., Gu B., Zhu H., Wang D. (2023). Association of KMT2C/D loss-of-function variants with response to immune checkpoint blockades in colorectal cancer. Cancer Sci..

[14] Lin Y., Luo S., Luo M., Lu X., Li Q., Xie M., Huang Y., Liao X., Zhang Y., Li Y. (2023). Homologous recombination repair gene mutations in colorectal cancer favors treatment of immune checkpoint inhibitors. Mol. Carcinog..

[15] Guinney J., Dienstmann R., Wang X., de Reyniès A., Schlicker A., Soneson C., Marisa L., Roepman P., Nyamundanda G., Angelino P. (2015). The consensus molecular subtypes of colorectal cancer. Nat. Med..

[16] Coussens L.M., Werb Z. (2002). Inflammation and cancer. Nature.

[17] Nathan C., Ding A. (2010). Nonresolving Inflammation. Cell.

[18] Zhao H., Wu L., Yan G., Chen Y., Zhou M., Wu Y., Li Y. (2021). Inflammation and tumor progression: Signaling pathways and targeted intervention. Signal Transduct. Target. Ther..

[19] Tuomisto A.E., Mäkinen M.J., Väyrynen J.P. (2019). Systemic inflammation in colorectal cancer: Underlying factors, effects, and prognostic significance. World J. Gastroenterol..

[20] Ma Y., Adjemian S., Mattarollo S.R., Yamazaki T., Aymeric L., Yang H., Catani J.P.P., Hannani D., Duret H., Steegh K. (2013). Anticancer Chemotherapy-Induced Intratumoral Recruitment and Differentiation of Antigen-Presenting Cells. Immunity.

[21] Bystrom J., Evans I., Newson J., Stables M., Toor I., van Rooijen N., Crawford M., Colville-Nash P., Farrow S., Gilroy D.W. (2008). Resolution-phase macrophages possess a unique inflammatory phenotype that is controlled by cAMP. Blood.

[22] Williams M.R., Azcutia V., Newton G., Alcaide P., Luscinskas F.W. (2011). Emerging mechanisms of neutrophil recruitment across endothelium. Trends Immunol..

[23] Nishida A., Andoh A. (2025). The Role of Inflammation in Cancer: Mechanisms of Tumor Initiation, Progression, and Metastasis. Cells.

[24] Ohue Y., Nishikawa H. (2019). Regulatory T (Treg) cells in cancer: Can Treg cells be a new therapeutic target?. Cancer Sci..

[25] Hickman B., Salonen A., Ponsero A.J., Jokela R., Kolho K.-L., de Vos W.M., Korpela K. (2024). Gut microbiota wellbeing index predicts overall health in a cohort of 1000 infants. Nat. Commun..

[26] Iebba V., Totino V., Gagliardi A., Santangelo F., Cacciotti F., Trancassini M., Mancini C., Cicerone C., Corazziari E., Pantanella F. (2016). Eubiosis and dysbiosis: The two sides of the microbiota. New Microbiol..

[27] Bai X., Wei H., Liu W., Coker O.O., Gou H., Liu C., Zhao L., Li C., Zhou Y., Wang G. (2022). Cigarette smoke promotes colorectal cancer through modulation of gut microbiota and related metabolites. Gut.

[28] Fackelmann G., Manghi P., Carlino N., Heidrich V., Piccinno G., Ricci L., Piperni E., Arrè A., Bakker E., Creedon A.C. (2025). Gut microbiome signatures of vegan, vegetarian and omnivore diets and associated health outcomes across 21,561 individuals. Nat. Microbiol..

[29] Feng Q., Liang S., Jia H., Stadlmayr A., Tang L., Lan Z., Zhang D., Xia H., Xu X., Jie Z. (2015). Gut microbiome development along the colorectal adenoma–carcinoma sequence. Nat. Commun..

[30] Kennedy M.S., Freiburger A., Cooper M., Beilsmith K., George M.L.S., Kalski M., Cham C., Guzzetta A., Ng S.C., Chan F.K. (2025). Diet outperforms microbial transplant to drive microbiome recovery in mice. Nature.

[31] Ramaboli M.C., Ocvirk S., Mirzaei M.K., Eberhart B.L., Valdivia-Garcia M., Metwaly A., Neuhaus K., Barker G., Ru J., Nesengani L.T. (2024). Diet changes due to urbanization in South Africa are linked to microbiome and metabolome signatures of Westernization and colorectal cancer. Nat. Commun..

[32] Qin Y., Havulinna A.S., Liu Y., Jousilahti P., Ritchie S.C., Tokolyi A., Sanders J.G., Valsta L., Brożyńska M., Zhu Q. (2022). Combined effects of host genetics and diet on human gut microbiota and incident disease in a single population cohort. Nat. Genet..

[33] Sobhani I., Tap J., Roudot-Thoraval F., Roperch J.P., Letulle S., Langella P., Corthier G., Van Nhieu J.T., Furet J.P. (2011). Microbial Dysbiosis in Colorectal Cancer (CRC) Patients. PLoS ONE.

[34] Thomas A.M., Manghi P., Asnicar F., Pasolli E., Armanini F., Zolfo M., Beghini F., Manara S., Karcher N., Pozzi C. (2019). Metagenomic analysis of colorectal cancer datasets identifies cross-cohort microbial diagnostic signatures and a link with choline degradation. Nat. Med..

[35] Wang T., Cai G., Qiu Y., Fei N., Zhang M., Pang X., Jia W., Cai S., Zhao L. (2011). Structural segregation of gut microbiota between colorectal cancer patients and healthy volunteers. ISME J..

[36] Wirbel J., Pyl P.T., Kartal E., Zych K., Kashani A., Milanese A., Fleck J.S., Voigt A.Y., Palleja A., Ponnudurai R. (2019). Meta-analysis of fecal metagenomes reveals global microbial signatures that are specific for colorectal cancer. Nat. Med..

[37] Yachida S., Mizutani S., Shiroma H., Shiba S., Nakajima T., Sakamoto T., Watanabe H., Masuda K., Nishimoto Y., Kubo M. (2019). Metagenomic and metabolomic analyses reveal distinct stage-specific phenotypes of the gut microbiota in colorectal cancer. Nat. Med..

[38] Yu J., Feng Q., Wong S.H., Zhang D., Liang Q.Y., Qin Y., Tang L., Zhao H., Stenvang J., Li Y. (2015). Metagenomic analysis of faecal microbiome as a tool towards targeted non-invasive biomarkers for colorectal cancer. Gut.

[39] Li L., Li X., Zhong W., Yang M., Xu M., Sun Y., Ma J., Liu T., Song X., Dong W. (2019). Gut microbiota from colorectal cancer patients enhances the progression of intestinal adenoma in Apcmin/+ mice. eBioMedicine.

[40] Wong S.H., Zhao L., Zhang X., Nakatsu G., Han J., Xu W., Xiao X., Kwong T.N.Y., Tsoi H., Wu W.K.K. (2017). Gavage of Fecal Samples From Patients With Colorectal Cancer Promotes Intestinal Carcinogenesis in Germ-Free and Conventional Mice. Gastroenterology.

[41] Dai Z., Coker O.O., Nakatsu G., Wu W.K.K., Zhao L., Chen Z., Chan F.K.L., Kristiansen K., Sung J.J.Y., Wong S.H. (2018). Multi-cohort analysis of colorectal cancer metagenome identified altered bacteria across populations and universal bacterial markers. Microbiome.

[42] Flemer B., Warren R.D., Barrett M.P., Cisek K., Das A., Jeffery I.B., Hurley E., O’Riordain M., Shanahan F., O’Toole P.W. (2018). The oral microbiota in colorectal cancer is distinctive and predictive. Gut.

[43] Long X., Wong C.C., Tong L., Chu E.S.H., Szeto C.H., Go M.Y.Y., Coker O.O., Chan A.W.H., Chan F.K.L., Sung J.J.Y. (2019). *Peptostreptococcus anaerobius* promotes colorectal carcinogenesis and modulates tumour immunity. Nat. Microbiol..

[44] Nakatsu G., Li X., Zhou H., Sheng J., Wong S.H., Wu W.K.K., Ng S.C., Tsoi H., Dong Y., Zhang N. (2015). Gut mucosal microbiome across stages of colorectal carcinogenesis. Nat. Commun..

[45] Zhou S.-H., Du Y., Xue W.-Q., He M.-J., Zhou T., Zhao Z.-Y., Pei L., Chen Y.-W., Xie J.-R., Huang C.-L. (2025). Oral microbiota signature predicts the prognosis of colorectal carcinoma. npj Biofilms Microbiomes.

[46] Aran V., Victorino A.P., Thuler L.C., Gil Ferreira C. (2016). Colorectal Cancer: Epidemiology, Disease Mechanisms and Interventions to Reduce Onset and Mortality. Clin. Color. Cancer.

[47] Van Blarigan E.L., Ma C., Ou F.S., Bainter T.M., Venook A.P., Ng K., Niedzwiecki D., Giovannucci E., Lenz H.J., Polite B.N. (2023). Dietary fat in relation to all-cause mortality and cancer progression and death among people with metastatic colorectal cancer: Data from CALGB 80405 (Alliance)/SWOG. Int. J. Cancer.

[48] Birt D.F., Phillips G.J. (2014). Diet, genes, and microbes: Complexities of colon cancer prevention. Toxicol. Pathol..

[49] Garavaglia B., Vallino L., Ferraresi A., Amoruso A., Pane M., Isidoro C. (2025). Probiotic-Derived Metabolites from *Lactiplantibacillus plantarum* OC01 Reprogram Tumor-Associated Macrophages to an Inflammatory Anti-Tumoral Phenotype: Impact on Colorectal Cancer Cell Proliferation and Migration. Biomedicines.

[50] Hanus M., Parada-Venegas D., Landskron G., Wielandt A.M., Hurtado C., Alvarez K., Hermoso M.A., López-Köstner F., De la Fuente M. (2021). Immune System, Microbiota, and Microbial Metabolites: The Unresolved Triad in Colorectal Cancer Microenvironment. Front. Immunol..

[51] Kachroo P., Ivanov I., Davidson L.A., Chowdhary B.P., Lupton J.R., Chapkin R.S. (2011). Classification of Diet-Modulated Gene Signatures at the Colon Cancer Initiation and Progression Stages. Dig. Dis. Sci..

[52] Amitay E.L., Krilaviciute A., Brenner H. (2018). Systematic review: Gut microbiota in fecal samples and detection of colorectal neoplasms. Gut Microbes.

[53] Wang Q., Hu T., Zhang Q., Zhang Y., Dong X., Jin Y., Li J., Guo Y., Guo F., Chen Z. (2024). *Fusobacterium nucleatum* promotes colorectal cancer through neogenesis of tumor stem cells. J. Clin. Investig..

[54] Li Z., Deng L., Cheng M., Ye X., Yang N., Fan Z., Sun L. (2025). Emerging role of bile acids in colorectal liver metastasis: From molecular mechanism to clinical significance (Review). Int. J. Oncol..

[55] Thanikachalam K., Khan G. (2019). Colorectal Cancer and Nutrition. Nutrients.

[56] Reglero C., Reglero G. (2019). Precision Nutrition and Cancer Relapse Prevention: A Systematic Literature Review. Nutrients.

[57] Pérez-Escalante E., Cariño-Cortés R., Fernández-Martínez E., Ortiz M.I., Muñoz-Pérez V.M., Sánchez-Crisóstomo I., Jiménez-Ángeles L. (2019). Colorectal Cancer: Causes and Evidence of Chemopreventive Treatments. Curr. Pharm. Biotechnol..

[58] Chen Y., Kong W., Liu M., Li Q., Wang Y., Zheng Y., Zhou Y. (2023). Metabolic syndrome and risk of colorectal cancer: A Mendelian randomization study. Heliyon.

[59] Mili N., Paschou S.A., Goulis D.G., Dimopoulos M.-A., Lambrinoudaki I., Psaltopoulou T. (2021). Obesity, metabolic syndrome, and cancer: Pathophysiological and therapeutic associations. Endocrine.

[60] Tomasello G., Mazzola M., Leone A., Sinagra E., Zummo G., Farina F., Damiani P., Cappello F., Geagea A.G., Jurjus A. (2016). Nutrition, oxidative stress and intestinal dysbiosis: Influence of diet on gut microbiota in inflammatory bowel diseases. Biomed. Pap..

[61] Ringel A.E., Drijvers J.M., Baker G.J., Catozzi A., García-Cañaveras J.C., Gassaway B.M., Miller B.C., Juneja V.R., Nguyen T.H., Joshi S. (2020). Obesity Shapes Metabolism in the Tumor Microenvironment to Suppress Anti-Tumor Immunity. Cell.

[62] Davis C., Bryan J., Hodgson J., Murphy K. (2015). Definition of the Mediterranean Diet; A Literature Review. Nutrients.

[63] Guasch-Ferré M., Willett W.C. (2021). The Mediterranean diet and health: A comprehensive overview. J. Intern. Med..

[64] Divella R., Daniele A., Savino E., Paradiso A. (2020). Anticancer Effects of Nutraceuticals in the Mediterranean Diet: An Epigenetic Diet Model. Cancer Genom.—Proteom..

[65] Calibasi-Kocal G., Pakdemirli A., Bayrak S., Ozupek N.M., Sever T., Basbinar Y., Ellidokuz H., Yigitbasi T. (2019). Curcumin effects on cell proliferation, angiogenesis and metastasis in colorectal cancer. J. BUON.

[66] Gallardo M., Kemmerling U., Aguayo F., Bleak T.C., Muñoz J.P., Calaf G.M. (2019). Curcumin rescues breast cells from epithelial-mesenchymal transition and invasion induced by anti-miR-34a. Int. J. Oncol..

[67] Pickich M.B., Hargrove M.W., Phillips C.N., Healy J.C., Moore A.N., Roberts M.D., Martin J.S. (2019). Effect of curcumin supplementation on serum expression of select cytokines and chemokines in a female rat model of nonalcoholic steatohepatitis. BMC Res. Notes.

[68] Tomeh M.A., Hadianamrei R., Zhao X. (2019). A Review of Curcumin and Its Derivatives as Anticancer Agents. Int. J. Mol. Sci..

[69] Weng W., Goel A. (2022). Curcumin and colorectal cancer: An update and current perspective on this natural medicine. Semin. Cancer Biol..

[70] Liu X.-F., Shao J.-H., Liao Y.-T., Wang L.-N., Jia Y., Dong P.-J., Liu Z.-Z., He D.-D., Li C., Zhang X. (2023). Regulation of short-chain fatty acids in the immune system. Front. Immunol..

[71] Zhang H., Tian Y., Xu C., Chen M., Xiang Z., Gu L., Xue H., Xu Q. (2025). Crosstalk between gut microbiotas and fatty acid metabolism in colorectal cancer. Cell Death Discov..

[72] Halestrap A.P., Meredith D. (2003). The SLC16 gene family—From monocarboxylate transporters (MCTs) to aromatic amino acid transporters and beyond. Pflügers Arch. Eur. J. Physiol..

[73] Islam R., Anzai N., Ahmed N., Ellapan B., Jin C.J., Srivastava S., Miura D., Fukutomi T., Kanai Y., Endou H. (2008). Mouse Organic Anion Transporter 2 (mOat2) Mediates the Transport of Short Chain Fatty Acid Propionate. J. Pharmacol. Sci..

[74] Kim C.H., Park J., Kim M. (2014). Gut Microbiota-Derived Short-Chain Fatty Acids, T Cells, and Inflammation. Immune Netw..

[75] Luu M., Visekruna A. (2019). Short-chain fatty acids: Bacterial messengers modulating the immunometabolism of T cells. Eur. J. Immunol..

[76] Moniri N.H., Farah Q. (2021). Short-chain free-fatty acid G protein-coupled receptors in colon cancer. Biochem. Pharmacol..

[77] Mann E.R., Lam Y.K., Uhlig H.H. (2024). Short-chain fatty acids: Linking diet, the microbiome and immunity. Nat. Rev. Immunol..

[78] Park J., Kim M., Kang S., Jannasch A., Cooper B., Patterson J., Kim C. (2014). Short-chain fatty acids induce both effector and regulatory T cells by suppression of histone deacetylases and regulation of the mTOR–S6K pathway. Mucosal Immunol..

[79] Bao X., Wu J. (2021). Impact of food-derived bioactive peptides on gut function and health. Food Res. Int..

[80] Nielsen S.D.-H., Liang N., Rathish H., Kim B.J., Lueangsakulthai J., Koh J., Qu Y., Schulz H.-J., Dallas D.C. (2023). Bioactive milk peptides: An updated comprehensive overview and database. Crit. Rev. Food Sci. Nutr..

[81] Hoskin D.W., Ramamoorthy A. (2008). Studies on anticancer activities of antimicrobial peptides. Biochim. Biophys. Acta (BBA)-Biomembr..

[82] Zhang Y., Wang C., Zhang W., Li X. (2023). Bioactive peptides for anticancer therapies. Biomater. Transl..

[83] Benigni A., Cassis P., Remuzzi G. (2010). Angiotensin II revisited: New roles in inflammation, immunology and aging. EMBO Mol. Med..

[84] Günther J., Kill A., Becker M.O., Heidecke H., Rademacher J., Siegert E., Radić M., Burmester G.-R., Dragun D., Riemekasten G. (2014). Angiotensin receptor type 1 and endothelin receptor type A on immune cells mediate migration and the expression of IL-8 and CCL18 when stimulated by autoantibodies from systemic sclerosis patients. Arthritis Res. Ther..

[85] Nakamura K., Yaguchi T., Ohmura G., Kobayashi A., Kawamura N., Iwata T., Kiniwa Y., Okuyama R., Kawakami Y. (2017). Involvement of local renin-angiotensin system in immunosuppression of tumor microenvironment. Cancer Sci..

[86] Maeda A., Okazaki T., Inoue M., Kitazono T., Yamasaki M., Lemonnier F.A., Ozaki S. (2009). Immunosuppressive effect of angiotensin receptor blocker on stimulation of mice CTLs by angiotensin II. Int. Immunopharmacol..

[87] Silva-Filho J.L., Caruso-Neves C., Pinheiro A.A.S. (2016). Angiotensin II type-1 receptor (AT1R) regulates expansion, differentiation, and functional capacity of antigen-specific CD8+ T cells. Sci. Rep..

[88] Barhoumi T., Mansour F.A., Jalouli M., Alamri H.S., Ali R., Harrath A.H., Aljumaa M., Boudjelal M. (2023). Angiotensin II modulates THP-1-like macrophage phenotype and inflammatory signatures via angiotensin II type 1 receptor. Front. Cardiovasc. Med..

[89] Mei J., Chu J., Yang K., Luo Z., Yang J., Xu J., Li Q., Zhang Y., Zhang Q., Wan M. (2024). Angiotensin receptor blocker attacks armored and cold tumors and boosts immune checkpoint blockade. J. Immunother. Cancer.

[90] Beitia M., Solano-Iturri J.D., Errarte P., Sanz B., Perez I., Etxezarraga M.C., Loizate A., Asumendi A., Larrinaga G. (2019). Altered expression of renin-angiotensin system receptors throughout colorectal adenoma-adenocarcinoma sequence. Int. J. Med. Sci..

[91] Hashemzehi M., Rahmani F., Khoshakhlagh M., Avan A., Asgharzadeh F., Barneh F., Moradi-Marjaneh R., Soleimani A., Fiuji H., Ferns G.A. (2021). Angiotensin receptor blocker Losartan inhibits tumor growth of colorectal cancer. EXCLI J..

[92] Vistoli G., De Maddis D., Cipak A., Zarkovic N., Carini M., Aldini G. (2013). Advanced glycoxidation and lipoxidation end products (AGEs and ALEs): An overview of their mechanisms of formation. Free Radic. Res..

[93] Arihara K., Zhou L., Ohata M. (2017). Bioactive Properties of Maillard Reaction Products Generated From Food Protein-derived Peptides. Adv. Food Nutr. Res..

[94] Muthyalaiah Y.S., Jonnalagadda B., John C.M., Arockiasamy S. (2021). Impact of Advanced Glycation End products (AGEs) and its receptor (RAGE) on cancer metabolic signaling pathways and its progression. Glycoconj. J..

[95] Nass N., Bartling B., Santos A.N., Scheubel R.J., Börgermann J., Silber R.E., Simm A. (2007). Advanced glycation end products, diabetes and ageing. Z. Gerontol. Geriatr..

[96] Shen C.-Y., Lu C.-H., Cheng C.-F., Li K.-J., Kuo Y.-M., Wu C.-H., Liu C.-H., Hsieh S.-C., Tsai C.-Y., Yu C.-L. (2024). Advanced Glycation End-Products Acting as Immunomodulators for Chronic Inflammation, Inflammaging and Carcinogenesis in Patients with Diabetes and Immune-Related Diseases. Biomedicines.

[97] Hofmann M.A., Drury S., Fu C., Qu W., Taguchi A., Lu Y., Avila C., Kambham N., Bierhaus A., Nawroth P. (1999). RAGE mediates a novel proinflammatory axis: A central cell surface receptor for S100/calgranulin polypeptides. Cell.

[98] Noriega D.B., Zenker H.E., Croes C.-A., Ewaz A., Ruinemans-Koerts J., Savelkoul H.F.J., van Neerven R.J.J., Teodorowicz M. (2022). Receptor Mediated Effects of Advanced Glycation End Products (AGEs) on Innate and Adaptative Immunity: Relevance for Food Allergy. Nutrients.

[99] Thornalley P.J. (1998). Cell activation by glycated proteins. AGE receptors, receptor recognition factors and functional classification of AGEs. Cell. Mol. Biol..

[100] Sakellariou S., Fragkou P., Levidou G., Gargalionis A.N., Piperi C., Dalagiorgou G., Adamopoulos C., Saetta A., Agrogiannis G., Theohari I. (2016). Clinical significance of AGE-RAGE axis in colorectal cancer: Associations with glyoxalase-I, adiponectin receptor expression and prognosis. BMC Cancer.

[101] Azizian-Farsani F., Abedpoor N., Sheikhha M.H., Gure A.O., Nasr-Esfahani M.H., Ghaedi K. (2020). Receptor for Advanced Glycation End Products Acts as a Fuel to Colorectal Cancer Development. Front. Oncol..

[102] Li Y., Wang J., Zhong S., Li J., Du W. (2020). Scutellarein inhibits the development of colon cancer via CDC4-mediated RAGE ubiquitination. Int. J. Mol. Med..

[103] Li J., Baker J.R., Aglago E.K., Zhao Z., Jiao L., Freisling H., Hughes D.J., Eriksen A.K., Tjønneland A., Severi G. (2024). Pre-diagnostic plasma advanced glycation end-products and soluble receptor for advanced glycation end-products and mortality in colorectal cancer patients. Int. J. Cancer.

[104] Mao Z., Aglago E.K., Zhao Z., Schalkwijk C., Jiao L., Freisling H., Weiderpass E., Hughes D.J., Eriksen A.K., Tjønneland A. (2021). Dietary Intake of Advanced Glycation End Products (AGEs) and Mortality among Individuals with Colorectal Cancer. Nutrients.

[105] Di Paola M., De Filippo C., Cavalieri D., Ramazzotti M., Poullet J., Massart S., Collini S., Pieraccini G., Lionetti P. (2011). PP90 IMPACT OF DIET IN SHAPING GUT MICROBIOTA REVEALED BY A COMPARATIVE STUDY IN CHILDREN FROM EUROPE AND RURAL AFRICA. Dig. Liver Dis..

[106] O’Keefe S.J.D., Li J.V., Lahti L., Ou J., Carbonero F., Mohammed K., Posma J.M., Kinross J., Wahl E., Ruder E. (2015). Fat, fibre and cancer risk in African Americans and rural Africans. Nat. Commun..

[107] Donohoe D.R., Collins L.B., Wali A., Bigler R., Sun W., Bultman S.J. (2012). The Warburg Effect Dictates the Mechanism of Butyrate-Mediated Histone Acetylation and Cell Proliferation. Mol. Cell.

[108] Donohoe D.R., Holley D., Collins L.B., Montgomery S.A., Whitmore A.C., Hillhouse A., Curry K.P., Renner S.W., Greenwalt A., Ryan E.P. (2014). A gnotobiotic mouse model demonstrates that dietary fiber protects against colorectal tumorigenesis in a microbiota- and butyrate-dependent manner. Cancer Discov..

[109] Kunzmann A.T., Coleman H.G., Huang W.-Y., Kitahara C.M., Cantwell M.M., Berndt S.I. (2015). Dietary fiber intake and risk of colorectal cancer and incident and recurrent adenoma in the Prostate, Lung, Colorectal, and Ovarian Cancer Screening Trial. Am. J. Clin. Nutr..

[110] Ocvirk S., Wilson A.S., Posma J.M., Li J.V., Koller K.R., Day G.M., Flanagan C.A., Otto J.E., Sacco P.E., Sacco F.D. (2020). A prospective cohort analysis of gut microbial co-metabolism in Alaska Native and rural African people at high and low risk of colorectal cancer. Am. J. Clin. Nutr..

[111] Vital M., Karch A., Pieper D.H. (2017). Colonic Butyrate-Producing Communities in Humans: An Overview Using Omics Data. mSystems.

[112] Cao H., Xu M., Dong W., Deng B., Wang S., Zhang Y., Wang S., Luo S., Wang W., Qi Y. (2017). Secondary bile acid-induced dysbiosis promotes intestinal carcinogenesis. Int. J. Cancer.

[113] Flynn C., Montrose D.C., Swank D.L., Nakanishi M., Ilsley J.N., Rosenberg D.W. (2006). Deoxycholic acid promotes the growth of colonic aberrant crypt foci. Mol. Carcinog..

[114] Weir T.L., Manter D.K., Sheflin A.M., Barnett B.A., Heuberger A.L., Ryan E.P. (2013). Stool Microbiome and Metabolome Differences between Colorectal Cancer Patients and Healthy Adults. PLoS ONE.

[115] Hanahan D., Weinberg R.A. (2011). Hallmarks of cancer: The next generation. Cell.

[116] Sedlak J.C., Yilmaz O.H., Roper J. (2023). Metabolism and Colorectal Cancer. Annu. Rev. Pathol..

[117] Sengupta S., Peterson T.R., Sabatini D.M. (2010). Regulation of the mTOR Complex 1 Pathway by Nutrients, Growth Factors, and Stress. Mol. Cell.

[118] Ahmad I., Hoque M., Alam S.S.M., Zughaibi T.A., Tabrez S. (2023). Curcumin and Plumbagin Synergistically Target the PI3K/Akt/mTOR Pathway: A Prospective Role in Cancer Treatment. Int. J. Mol. Sci..

[119] He Q., Liu C., Wang X., Rong K., Zhu M., Duan L., Zheng P., Mi Y. (2023). Exploring the mechanism of curcumin in the treatment of colon cancer based on network pharmacology and molecular docking. Front. Pharmacol..

[120] Ali M.U., Ur Rahman M.S., Jia Z., Jiang C. (2017). Eukaryotic translation initiation factors and cancer. Tumor Biol..

[121] Golob-Schwarzl N., Schweiger C., Koller C., Krassnig S., Gogg-Kamerer M., Gantenbein N., Toeglhofer A.M., Wodlej C., Bergler H., Pertschy B. (2017). Separation of low and high grade colon and rectum carcinoma by eukaryotic translation initiation factors 1, 5 and 6. Oncotarget.

[122] Rosenwald I.B., Chen J.-J., Wang S., Savas L., London I.M., Pullman J. (1999). Upregulation of protein synthesis initiation factor eIF-4E is an early event during colon carcinogenesis. Oncogene.

[123] Bordone R., Ivy D.M., D’aMico R., Barba M., Gaggianesi M., Di Pastena F., Cesaro B., Bufalieri F., Balzerano A., De Smaele E. (2024). MYC upstream region orchestrates resistance to PI3K inhibitors in cancer cells through FOXO3a-mediated autophagic adaptation. Oncogene.

[124] Kreuzaler P., Inglese P., Ghanate A., Gjelaj E., Wu V., Panina Y., Mendez-Lucas A., MacLachlan C., Patani N., Hubert C.B. (2023). Vitamin B5 supports MYC oncogenic metabolism and tumor progression in breast cancer. Nat. Metab..

[125] Wang H., Sun J., Sun H., Wang Y., Lin B., Wu L., Qin W., Zhu Q., Yi W. (2024). The OGT–c-Myc–PDK2 axis rewires the TCA cycle and promotes colorectal tumor growth. Cell Death Differ..

[126] Scheper G., Voorma H., Thomas A. (1992). Eukaryotic initiation factors-4E and -4F stimulate 5’ cap-dependent as well as internal initiation of protein synthesis. J. Biol. Chem..

[127] Chiu H., Jackson L.V., Oh K.I., Mai A., Ronai Z.A., Ruggero D., Fruman D.A. (2019). The mTORC1/4E-BP/eIF4E Axis Promotes Antibody Class Switching in B Lymphocytes. J. Immunol..

[128] So L., Lee J., Palafox M., Mallya S., Woxland C.G., Arguello M., Truitt M.L., Sonenberg N., Ruggero D., Fruman D.A. (2016). The 4E-BP–eIF4E axis promotes rapamycin-sensitive growth and proliferation in lymphocytes. Sci. Signal..

[129] Mao X., Green J., Safer B., Lindsten T., Frederickson R., Miyamoto S., Sonenberg N., Thompson C. (1992). Regulation of translation initiation factor gene expression during human T cell activation. J. Biol. Chem..

[130] Chen J., Ye J., Lai R. (2023). A lipid metabolism-related gene signature reveals dynamic immune infiltration of the colorectal adenoma-carcinoma sequence. Lipids Health Dis..

[131] Markowski A.R., Błachnio-Zabielska A.U., Pogodzińska K., Markowska A.J., Zabielski P. (2023). Diverse Sphingolipid Profiles in Rectal and Colon Cancer. Int. J. Mol. Sci..

[132] Nicolini A., Ferrari P. (2024). Involvement of tumor immune microenvironment metabolic reprogramming in colorectal cancer progression, immune escape, and response to immunotherapy. Front. Immunol..

[133] He J., Chai X., Zhang Q., Wang Y., Wang Y., Yang X., Wu J., Feng B., Sun J., Rui W. (2025). The lactate receptor HCAR1 drives the recruitment of immunosuppressive PMN-MDSCs in colorectal cancer. Nat. Immunol..

[134] Watson M.J., Vignali P.D.A., Mullett S.J., Overacre-Delgoffe A.E., Peralta R.M., Grebinoski S., Menk A.V., Rittenhouse N.L., DePeaux K., Whetstone R.D. (2021). Metabolic support of tumour-infiltrating regulatory T cells by lactic acid. Nature.

[135] Zhang D., Tang Z., Huang H., Zhou G., Cui C., Weng Y., Liu W., Kim S., Lee S., Perez-Neut M. (2019). Metabolic regulation of gene expression by histone lactylation. Nature.

[136] Zhou J., Xu W., Wu Y., Wang M., Zhang N., Wang L., Feng Y., Zhang T., Wang L., Mao A. (2023). GPR37 promotes colorectal cancer liver metastases by enhancing the glycolysis and histone lactylation via Hippo pathway. Oncogene.

[137] Li X.-M., Yang Y., Jiang F.-Q., Hu G., Wan S., Yan W.-Y., He X.-S., Xiao F., Yang X.-M., Guo X. (2024). Histone lactylation inhibits RARγ expression in macrophages to promote colorectal tumorigenesis through activation of TRAF6-IL-6-STAT3 signaling. Cell Rep..

[138] Gu J., Xu X., Li X., Yue L., Zhu X., Chen Q., Gao J., Takashi M., Zhao W., Zhao B. (2024). Tumor-resident microbiota contributes to colorectal cancer liver metastasis by lactylation and immune modulation. Oncogene.

[139] Bai Y., Li T., Wang Q., You W., Yang H., Xu X., Li Z., Zhang Y., Yan C., Yang L. (2024). Shaping immune landscape of colorectal cancer by cholesterol metabolites. EMBO Mol. Med..

[140] Wu F., Feng Z., Wang X., Guo Y., Wu B., Bai S., Lan N., Chen M., Ren J. (2025). Sphingosine-1-phosphate stimulates colorectal cancer tumor microenvironment angiogenesis and induces macrophage polarization via macrophage migration inhibitory factor. Front. Immunol..

[141] Zhu M., Hu Y., Gu Y., Lin X., Jiang X., Gong C., Fang Z. (2025). Role of amino acid metabolism in tumor immune microenvironment of colorectal cancer. Am. J. Cancer Res..

[142] Brandacher G., Perathoner A., Ladurner R., Schneeberger S., Obrist P., Winkler C., Werner E.R., Werner-Felmayer G., Weiss H.G., G√∂Bel G. (2006). Prognostic value of indoleamine 2,3-dioxygenase expression in colorectal cancer: Effect on tumor-infiltrating T cells. Clin. Cancer Res..

[143] Stone T.W., Williams R.O. (2023). Modulation of T cells by tryptophan metabolites in the kynurenine pathway. Trends Pharmacol. Sci..

[144] Yu T., Van der Jeught K., Zhu H., Zhou Z., Sharma S., Liu S., Eyvani H., So K.M., Singh N., Wang J. (2024). Inhibition of Glutamate-to-Glutathione Flux Promotes Tumor Antigen Presentation in Colorectal Cancer Cells. Adv. Sci..

[145] Strakhova R., Cadassou O., Cros-Perrial E., Jordheim L.P. (2020). Regulation of tumor infiltrated innate immune cells by adenosine. Purinergic Signal..

[146] Pantel K., Alix-Panabières C. (2010). Circulating tumour cells in cancer patients: Challenges and perspectives. Trends Mol. Med..

[147] Adler A., Geiger S., Keil A., Bias H., Schatz P., Devos T., Dhein J., Zimmermann M., Tauber R., Wiedenmann B. (2014). Improving compliance to colorectal cancer screening using blood and stool based tests in patients refusing screening colonoscopy in Germany. BMC Gastroenterol..

[148] Liang P.S., Zaman A., Kaminsky A., Cui Y., Castillo G., Tenner C.T., Sherman S.E., Dominitz J.A. (2023). Blood Test Increases Colorectal Cancer Screening in Persons Who Declined Colonoscopy and Fecal Immunochemical Test: A Randomized Controlled Trial. Clin. Gastroenterol. Hepatol..

[149] Mannucci A., Goel A. (2024). Stool and blood biomarkers for colorectal cancer management: An update on screening and disease monitoring. Mol. Cancer.

[150] Cohen S.J.A., Punt C.J., Iannotti N., Saidman B.H., Sabbath K.D., Gabrail N.Y., Picus J., Morse M., Mitchell E., Miller M.C. (2008). Relationship of Circulating Tumor Cells to Tumor Response, Progression-Free Survival, and Overall Survival in Patients With Metastatic Colorectal Cancer. J. Clin. Oncol..

[151] Nakamura Y., Taniguchi H., Ikeda M., Bando H., Kato K., Morizane C., Esaki T., Komatsu Y., Kawamoto Y., Takahashi N. (2020). Clinical utility of circulating tumor DNA sequencing in advanced gastrointestinal cancer: SCRUM-Japan GI-SCREEN and GOZILA studies. Nat. Med..

[152] Born J., Hendricks A., Hauser C., Egberts J.-H., Becker T., Röder C., Sebens S. (2021). Detection of Marker Associated with CTC in Colorectal Cancer in Mononuclear Cells of Patients with Benign Inflammatory Intestinal Diseases. Cancers.

[153] Sun Q., Pastor L., Du J., Powell M.J., Zhang A., Bodmer W., Wu J., Zheng S., Sha M.Y. (2021). A novel xenonucleic acid-mediated molecular clamping technology for early colorectal cancer screening. PLoS ONE.

[154] Kinde I., Wu J., Papadopoulos N., Kinzler K.W., Vogelstein B. (2011). Detection and quantification of rare mutations with massively parallel sequencing. Proc. Natl. Acad. Sci. USA.

[155] Tamburini E., De Vita F., Ciardiello D., Bielo L.B., Napolitano S., Martinelli E., Troiani T., Nicastro A., Latiano T.P., Parente P. (2024). Comprehensive genomic profiling by liquid biopsy captures tumor heterogeneity and identifies cancer vulnerabilities in patients with RAS/BRAF wild-type metastatic colorectal cancer in the CAPRI 2-GOIM trial. Ann. Oncol..

[156] Chung D.C., Gray D.M., Singh H., Issaka R.B., Raymond V.M., Eagle C., Hu S., Chudova D.I., Talasaz A., Greenson J.K. (2024). A Cell-free DNA Blood-Based Test for Colorectal Cancer Screening. N. Engl. J. Med..

[157] Nakamura Y., Ozaki H., Ueno M., Komatsu Y., Yuki S., Esaki T., Taniguchi H., Sunakawa Y., Yamaguchi K., Kato K. (2025). Targeted therapy guided by circulating tumor DNA analysis in advanced gastrointestinal tumors. Nat. Med..

[158] Nakamura Y., Tsukada Y., Matsuhashi N., Murano T., Shiozawa M., Takahashi Y., Oki E., Goto M., Kagawa Y., Kanazawa A. (2024). Colorectal Cancer Recurrence Prediction Using a Tissue-Free Epigenomic Minimal Residual Disease Assay. Clin. Cancer Res..

[159] Engell H.C. (1955). Cancer cells in the circulating blood; a clinical study on the occurrence of cancer cells in the peripheral blood and in venous blood draining the tumour area at operation. Acta Chir. Scand. Suppl..

[160] Allard W.J., Matera J., Miller M.C., Repollet M., Connelly M.C., Rao C., Tibbe A.G.J., Uhr J.W., Terstappen L.W.M.M. (2004). Tumor cells circulate in the peripheral blood of all major carcinomas but not in healthy subjects or patients with nonmalignant diseases. Clin. Cancer Res..

[161] Li C., Li J. (2024). Dysregulation of systemic immunity in colorectal cancer and its clinical applications as biomarkers and therapeutics. Crit. Rev. Oncol..

[162] Loyon R., Jary M., Salomé B., Gomez-Cadena A., Galaine J., Kroemer M., Romero P., Trabanelli S., Adotévi O., Borg C. (2019). Peripheral Innate Lymphoid Cells Are Increased in First Line Metastatic Colorectal Carcinoma Patients: A Negative Correlation With Th1 Immune Responses. Front. Immunol..

[163] Shevchenko I., Serban D., Simion L., Motofei I., Cristea B.M., Dumitrescu D., Tudor C., Dascalu A.M., Serboiu C., Tribus L.C. (2025). Clinical Significance of Blood Cell-Derived Inflammation Markers in Assessing Potential Early and Late Postoperative Complications in Patients with Colorectal Cancer: A Systematic Review. J. Clin. Med..

[164] Avram L., Crișan D., Moldovan R.-C., Bogos L.-G., Iuga C.-A., Andraș D., Crișan S., Bodolea C., Nemeş A., Donca V. (2025). Metabolomic Exploration of Colorectal Cancer Through Amino Acids and Acylcarnitines Profiling of Serum Samples. Cancers.

[165] Tan B., Qiu Y., Zou X., Chen T., Xie G., Cheng Y., Dong T., Zhao L., Feng B., Hu X. (2013). Metabonomics Identifies Serum Metabolite Markers of Colorectal Cancer. J. Proteome Res..

[166] Abu Bakar M.F., Nawi A.M., Chin S.F., Makpol S. (2023). Current status of serum metabolites biomarkers for polyps and colorectal cancer: A systematic review. Gastroenterol. Rep..

[167] Benson A.B., Venook A.P., Adam M., Chang G., Chen Y.-J., Ciombor K.K., Cohen S.A., Cooper H.S., Deming D., Garrido-Laguna I. (2024). Colon Cancer, Version 3.2024, NCCN Clinical Practice Guidelines in Oncology. J. Natl. Compr. Cancer Netw..

[168] Wang F., Chen G., Zhang Z., Yuan Y., Wang Y., Gao Y., Sheng W., Wang Z., Li X., Yuan X. (2024). The Chinese Society of Clinical Oncology (CSCO): Clinical guidelines for the diagnosis and treatment of colorectal cancer, 2024 update. Cancer Commun..

[169] Mikalonis M., Avlund T.H., Løve U.S. (2024). Danish guidelines for treating acute colonic obstruction caused by colorectal cancer—A review. Front. Surg..

[170] Martinez C.A.R., Campos F.G. (2024). Current guidelines for the management of rectal cancer patients: A review of recent advances and strategies. Front. Public Health.

[171] Benson A.B., Venook A.P., Adam M., Chang G., Chen Y.-J., Ciombor K.K., Cohen S.A., Cooper H.S., Deming D., Garrido-Laguna I. (2024). NCCN Guidelines^®^ Insights: Rectal Cancer, Version 3.2024. J. Natl. Compr. Cancer Netw..

[172] Cotte E., Arquilliere J., Artru P., Bachet J.B., Benhaim L., Bibeau F., Christou N., Conroy T., Doyen J., Hoeffel C. (2025). Rectal cancer—French intergroup clinical practice guidelines for diagnosis, treatment, and follow-up (TNCD, SNFGE, FFCD, GERCOR, UNICANCER, SFCD, SFED, SFRO, ACHBT, SFP, RENAPE, SNFCP, AFEF, SFR, and GRECCAR). Dig. Liver Dis..

[173] Hunger R.M., Kowalski C., Paasch C., Kirbach J., Mantke R. (2024). Outcome variation and the role of caseload in certified colorectal cancer centers—A retrospective cohort analysis of 90 000 cases. Int. J. Surg..

[174] Habr-Gama A., de Souza P.M., Ribeiro U., Nadalin W., Gansl R., Sousa A.H., Campos F.G., Gama-Rodrigues J. (1998). Low rectal cancer: Impact of radiation and chemotherapy on surgical treatment. Dis. Colon. Rectum.

[175] Cassidy J., Tabernero J., Twelves C., Brunet R., Butts C., Conroy T., Debraud F., Figer A., Grossmann J., Sawada N. (2004). XELOX (Capecitabine Plus Oxaliplatin): Active First-Line Therapy for Patients With Metastatic Colorectal Cancer. J. Clin. Oncol..

[176] Colucci G., Gebbia V., Paoletti G., Giuliani F., Caruso M., Gebbia N., Cartenì G., Agostara B., Pezzella G., Manzione L. (2005). Phase III Randomized Trial of FOLFIRI Versus FOLFOX4 in the Treatment of Advanced Colorectal Cancer: A Multicenter Study of the Gruppo Oncologico Dell’Italia Meridionale. J. Clin. Oncol..

[177] Ducreux M., Bennouna J., Hebbar M., Ychou M., Lledo G., Conroy T., Adenis A., Faroux R., Rebischung C., Bergougnoux L. (2010). Capecitabine plus oxaliplatin (XELOX) versus 5-fluorouracil/leucovorin plus oxaliplatin (FOLFOX-6) as first-line treatment for metastatic colorectal cancer. Int. J. Cancer.

[178] Goldberg R.M., Sargent D.J., Morton R.F., Fuchs C.S., Ramanathan R.K., Williamson S.K., Findlay B.P., Pitot H.C., Alberts S.R. (2004). A Randomized Controlled Trial of Fluorouracil Plus Leucovorin, Irinotecan, and Oxaliplatin Combinations in Patients With Previously Untreated Metastatic Colorectal Cancer. J. Clin. Oncol..

[179] Zhao G., Gao P., Yang K.H., Tian J.H., Ma B. (2010). Capecitabine/oxaliplatin as first-line treatment for metastatic colorectal cancer: A meta-analysis. Color. Dis..

[180] Habiba K., Aziz K., Sanders K., Santiago C.M., Mahadevan L.S.K., Makarov V., Weiner B.R., Morell G., Krishnan S. (2019). Enhancing Colorectal Cancer Radiation Therapy Efficacy using Silver Nanoprisms Decorated with Graphene as Radiosensitizers. Sci. Rep..

[181] Agarwal A., Chang G.J., Hu C., Taggart M., Rashid A., Park I.J., You Y.N., Das P., Krishnan S., Crane C.H. (2013). Quantified pathologic response assessed as residual tumor burden is a predictor of recurrence-free survival in patients with rectal cancer who undergo resection after neoadjuvant chemoradiotherapy. Cancer.

[182] Noronha M.M., Almeida L.F.C., Cappellaro A.P., da Silva L.F.L., da Conceição L.D., de Menezes J.S.A., Belotto M., Peixoto R.D. (2025). Neoadjuvant chemotherapy for colon cancer: A systematic review and meta-analysis of randomized controlled trials. Eur. J. Cancer.

[183] Bach S.P., Gilbert A., Brock K., Korsgen S., Geh I., Hill J., Gill T., Hainsworth P., Tutton M.G., Khan J. (2021). Radical surgery versus organ preservation via short-course radiotherapy followed by transanal endoscopic microsurgery for early-stage rectal cancer (TREC): A randomised, open-label feasibility study. Lancet Gastroenterol. Hepatol..

[184] Chai M., Wang S., Chen Y., Pei X., Zhen X. (2025). Targeted and intelligent nano-drug delivery systems for colorectal cancer treatment. Front. Bioeng. Biotechnol..

[185] Kumar A., Vaiphei K.K., Singh N., Chigurupati S.P.D., Paliwal S.R., Paliwal R., Gulbake A. (2024). Nanomedicine for colon-targeted drug delivery: Strategies focusing on inflammatory bowel disease and colon cancer. Nanomedicine.

[186] Tian S., Chen M. (2024). Global research progress of nanomedicine and colorectal cancer: A bibliometrics and visualization analysis. Front. Oncol..

[187] Zhang Z., Kuo J.C.-T., Zhang C., Huang Y., Lee R.J. (2022). Ivermectin Enhanced Antitumor Activity of Resiquimod in a Co-Loaded Squalene Emulsion. J. Pharm. Sci..

[188] Zhang Z., Kuo J.C.-T., Zhang C., Huang Y., Zhou Z., Lee R.J. (2021). A Squalene-Based Nanoemulsion for Therapeutic Delivery of Resiquimod. Pharmaceutics.

[189] McLaughlin M., Patin E.C., Pedersen M., Wilkins A., Dillon M.T., Melcher A.A., Harrington K.J. (2020). Inflammatory microenvironment remodelling by tumour cells after radiotherapy. Nat. Rev. Cancer.

[190] Jiang W., Chan C.K., Weissman I.L., Kim B.Y., Hahn S.M. (2016). Immune Priming of the Tumor Microenvironment by Radiation. Trends Cancer.

[191] Azeredo-Da-Silva A.L.F., de Jesus V.H.F., Agirrezabal I., Brennan V.K., Carion P.L., Amoury N., Vetromilla B.M., Zanotto B.S., Shergill S., Ziegelmann P.K. (2024). Selective Internal Radiation Therapy Using Y-90 Resin Microspheres for Metastatic Colorectal Cancer: An Updated Systematic Review and Network Meta-Analysis. Adv. Ther..

[192] Too C.W., Liang P.-C., Chotipanich C., Venkatanarashima N., Lo R.H., Da Zhuang K., Tan Z., Gogna A., Ng D.C.-E., Loke K.S.-H. (2025). Real-world outcomes of selective internal radiation therapy with ^90^Y resin microspheres in HCC/mCRC: RESIN registry Asia. Futur. Oncol..

[193] Lukovic J., Dawson L.A. (2024). Stereotactic body radiation therapy for colorectal cancer liver metastases. J. Gastrointest. Oncol..

[194] Underwood P.W., Ruff S.M., Pawlik T.M. (2024). Update on Targeted Therapy and Immunotherapy for Metastatic Colorectal Cancer. Cells.

[195] Thomas E.M., Wright J.A., Blake S.J., Page A.J., Worthley D.L., Woods S.L. (2023). Advancing translational research for colorectal immuno-oncology. Br. J. Cancer.

[196] Yu B., Kang J., Lei H., Li Z., Yang H., Zhang M. (2024). Immunotherapy for colorectal cancer. Front. Immunol..

[197] Ciracì P., Studiale V., Taravella A., Antoniotti C., Cremolini C. (2024). Late-line options for patients with metastatic colorectal cancer: A review and evidence-based algorithm. Nat. Rev. Clin. Oncol..

[198] Pizzamiglio M., Soulabaille A., Lahlou W., Pilla L., Zaanan A., Taieb J. (2025). Advances and challenges in targeted therapies for HER2-amplified colorectal cancer. Eur. J. Cancer.

[199] Dakowicz D., Zajkowska M., Mroczko B. (2022). Relationship between VEGF Family Members, Their Receptors and Cell Death in the Neoplastic Transformation of Colorectal Cancer. Int. J. Mol. Sci..

[200] Tampellini M., Sonetto C., Scagliotti G.V. (2016). Novel anti-angiogenic therapeutic strategies in colorectal cancer. Expert Opin. Investig. Drugs.

[201] Johnson R.M., Qu X., Lin C.-F., Huw L.-Y., Venkatanarayan A., Sokol E., Ou F.-S., Ihuegbu N., Zill O.A., Kabbarah O. (2022). ARID1A mutations confer intrinsic and acquired resistance to cetuximab treatment in colorectal cancer. Nat. Commun..

[202] Syaj S., Saeed A. (2024). Profile of Fruquintinib in the Management of Advanced Refractory Metastatic Colorectal Cancer: Design, Development and Potential Place in Therapy. Drug Des. Dev. Ther..

[203] de Gramont A., Van Cutsem E., Schmoll H.J., Tabernero J., Clarke S., Moore M.J., Cunningham D., Cartwright T.H., Hecht J.R., Rivera F. (2012). Bevacizumab plus oxaliplatin-based chemotherapy as adjuvant treatment for colon cancer (AVANT): A phase 3 randomised controlled trial. Lancet Oncol..

[204] Ott P.A., Hu-Lieskovan S., Chmielowski B., Govindan R., Naing A., Bhardwaj N., Margolin K., Awad M.M., Hellmann M.D., Lin J.J. (2020). A Phase Ib Trial of Personalized Neoantigen Therapy Plus Anti-PD-1 in Patients with Advanced Melanoma, Non-small Cell Lung Cancer, or Bladder Cancer. Cell.

[205] Chen E., Zhou W. (2025). Immunotherapy in microsatellite-stable colorectal cancer: Strategies to overcome resistance. Crit. Rev. Oncol..

[206] Han Y.-J., Shao C.-Y., Yao Y., Zhang Z., Fang M.-Z., Gong T., Zhang Y.-J., Li M. (2024). Immunotherapy of microsatellite stable colorectal cancer: Resistance mechanisms and treatment strategies. Postgrad. Med. J..

[207] Yap T.A., Bessudo A., Hamilton E., Sachdev J., Patel M.R., Rodon J., Evilevitch L., Duncan M., Guo W., Kumar S. (2022). IOLite: Phase 1b trial of doublet/triplet combinations of dostarlimab with niraparib, carboplatin–paclitaxel, with or without bevacizumab in patients with advanced cancer. J. Immunother. Cancer.

[208] Pum K., LaRocca C.J., Goffredo P., Subramanian S., Lou E., Prakash A. (2025). Immune Checkpoint Blockade Therapies for Colorectal Cancer: Current Strategies and Emerging Approaches. JCO Oncol. Adv..

[209] Le D.T., Diaz L.A., Kim T.W., Van Cutsem E., Geva R., Jäger D., Hara H., Burge M., O’Neil B.H., Kavan P. (2023). Pembrolizumab for previously treated, microsatellite instability–high/mismatch repair–deficient advanced colorectal cancer: Final analysis of KEYNOTE-164. Eur. J. Cancer.

[210] Diaz L.A., Shiu K.-K., Kim T.-W., Jensen B.V., Jensen L.H., Punt C., Smith D., Garcia-Carbonero R., Benavides M., Gibbs P. (2022). Pembrolizumab versus chemotherapy for microsatellite instability-high or mismatch repair-deficient metastatic colorectal cancer (KEYNOTE-177): Final analysis of a randomised, open-label, phase 3 study. Lancet Oncol..

[211] Casak S.J., Marcus L., Fashoyin-Aje L., Mushti S.L., Cheng J., Shen Y.-L., Pierce W.F., Her L., Goldberg K.B., Theoret M.R. (2021). FDA Approval Summary: Pembrolizumab for the First-line Treatment of Patients with MSI-H/dMMR Advanced Unresectable or Metastatic Colorectal Carcinoma. Clin. Cancer Res..

[212] Bever K.M., Durham J.N., Qi H., Azad N.S., Laheru D., Fisher G.A., Goldberg R.M., Greten T.F., Hays J.L., Krishnamurthy A. (2025). 10-year follow up of a phase 2 clinical trial of pembrolizumab (pembro) in microsatellite instability-high (MSI-H)/mismatch repair deficient (dMMR) advanced solid tumors. J. Clin. Oncol..

[213] Lenz H.-J., Overman M.J., Van Cutsem E., Limon M.L., Wong K.Y.M., Hendlisz A., Aglietta M., Garcia-Alfonso P., Neyns B., Gelsomino F. (2024). First-line (1L) nivolumab (NIVO) + ipilimumab (IPI) in patients (pts) with microsatellite instability-high/mismatch repair deficient (MSI-H/dMMR) metastatic colorectal cancer (mCRC): 64-month (mo) follow-up from CheckMate 142. J. Clin. Oncol..

[214] Chalabi M., Verschoor Y.L., Tan P.B., Balduzzi S., Van Lent A.U., Grootscholten C., Dokter S., Büller N.V., Grotenhuis B.A., Kuhlmann K. (2024). Neoadjuvant Immunotherapy in Locally Advanced Mismatch Repair–Deficient Colon Cancer. N. Engl. J. Med..

[215] Cercek A., Sinopoli J.C., Shia J., Weiss J.A., Temple L., Smith J.J., Saltz L.B., Widmar M., Fumo G., Aparo S. (2024). Durable complete responses to PD-1 blockade alone in mismatch repair deficient locally advanced rectal cancer. J. Clin. Oncol..

[216] Zhang X., Wu T., Cai X., Dong J., Xia C., Zhou Y., Ding R., Yang R., Tan J., Zhang L. (2022). Neoadjuvant Immunotherapy for MSI-H/dMMR Locally Advanced Colorectal Cancer: New Strategies and Unveiled Opportunities. Front. Immunol..

[217] Lenz H.-J., Van Cutsem E., Limon M.L., Wong K.Y.M., Hendlisz A., Aglietta M., García-Alfonso P., Neyns B., Luppi G., Cardin D.B. (2022). First-Line Nivolumab Plus Low-Dose Ipilimumab for Microsatellite Instability-High/Mismatch Repair-Deficient Metastatic Colorectal Cancer: The Phase II CheckMate 142 Study. J. Clin. Oncol..

[218] Overman M.J., McDermott R., Leach J.L., Lonardi S., Lenz H.-J., Morse M.A., Desai J., Hill A., Axelson M., Moss R.A. (2017). Nivolumab in patients with metastatic DNA mismatch repair-deficient or microsatellite instability-high colorectal cancer (CheckMate 142): An open-label, multicentre, phase 2 study. Lancet Oncol..

[219] Fukuoka S., Hara H., Takahashi N., Kojima T., Kawazoe A., Asayama M., Yoshii T., Kotani D., Tamura H., Mikamoto Y. (2020). Regorafenib Plus Nivolumab in Patients With Advanced Gastric or Colorectal Cancer: An Open-Label, Dose-Escalation, and Dose-Expansion Phase Ib Trial (REGONIVO, EPOC1603). J. Clin. Oncol..

[220] Antoniotti C., Borelli B., Rossini D., Pietrantonio F., Morano F., Salvatore L., Lonardi S., Marmorino F., Tamberi S., Corallo S. (2020). AtezoTRIBE: A randomised phase II study of FOLFOXIRI plus bevacizumab alone or in combination with atezolizumab as initial therapy for patients with unresectable metastatic colorectal cancer. BMC Cancer.

[221] Tran E., Robbins P.F., Lu Y.-C., Prickett T.D., Gartner J.J., Jia L., Pasetto A., Zheng Z., Ray S., Groh E.M. (2016). T-Cell Transfer Therapy Targeting Mutant KRAS in Cancer. N. Engl. J. Med..

[222] de Torres C.S., Elias E., Vaghi C., González N.S., García A., Alcaraz A., Rodríguez-Castells M., Baraibar I., Ros J., Salvà F. (2026). Exploring resistance to immune checkpoints inhibitors in mismatch repair-deficient or microsatellite-instable colorectal cancer. Cancer Treat. Rev..

[223] Hamid M.A., Pammer L.M., Lentner T.K., Doleschal B., Gruber R., Kocher F., Gasser E., Jöbstl A., Seeber A., Amann A. (2024). Immunotherapy for Microsatellite-Stable Metastatic Colorectal Cancer: Can we close the Gap between Potential and Practice?. Curr. Oncol. Rep..

[224] Wang K., Lou Y., Tao Z. (2024). A New Genetic Signature of Lactate Metabolism-Associated Genes Predicting Clinically Distinctive Features and Tumor Microenvironment in Colorectal Cancer. Cancer Control.

[225] Zhang E., Zhou X., Fan X., Li S., Ding C., Hong H., Aikemu B., Yang G., Yesseyeva G., Yang X. (2023). Exploring the relationship between lactate metabolism and immunological function in colorectal cancer through genes identification and analysis. Front. Cell Dev. Biol..

[226] Huang Y., Zhou J., Zhong H., Xie N., Zhang F.-R., Zhang Z. (2022). Identification of a novel lipid metabolism-related gene signature for predicting colorectal cancer survival. Front. Genet..

[227] Yang C., Huang S., Cao F., Zheng Y. (2021). A lipid metabolism-related genes prognosis biomarker associated with the tumor immune microenvironment in colorectal carcinoma. BMC Cancer.

[228] Huang X., Sun Y., Song J., Huang Y., Shi H., Qian A., Cao Y., Zhou Y., Wang Q. (2023). Prognostic value of fatty acid metabolism-related genes in colorectal cancer and their potential implications for immunotherapy. Front. Immunol..

[229] Zhang H., Cheng W., Zhao H., Chen W., Zhang Q., Yu Q.-Q. (2024). Identification and validation of novel prognostic fatty acid metabolic gene signatures in colon adenocarcinoma through systematic approaches. Oncol. Res. Featur. Preclin. Clin. Cancer Ther..

[230] Du F., Wu X., He Y., Zhao S., Xia M., Zhang B., Tong J., Xia T. (2024). Identification of an Amino Acid Metabolism Reprogramming Signature for Predicting Prognosis, Immunotherapy Efficacy, and Drug Candidates in Colon Cancer. Appl. Biochem. Biotechnol..

[231] Peng X., Zheng T., Guo Y., Zhu Y. (2022). Amino acid metabolism genes associated with immunotherapy responses and clinical prognosis of colorectal cancer. Front. Mol. Biosci..

[232] Shen K., Zhu C., Wu J., Yan J., Li P., Cao S., Zhou X., Yao G. (2024). Exploiting branched-chain amino acid metabolism and NOTCH3 expression to predict and target colorectal cancer progression. Front. Immunol..

[233] Li D., Cao D., Zhang Y., Yu X., Wu Y., Jia Z., Jiang J., Cao X. (2025). Integrative pan-cancer analysis and experiment validation identified GLS as a biomarker in tumor progression, prognosis, immune microenvironment, and immunotherapy. Sci. Rep..

[234] Xie Y., Li J., Tao Q., Wu Y., Liu Z., Zeng C., Chen Y. (2024). Identification of glutamine metabolism-related gene signature to predict colorectal cancer prognosis. J. Cancer.

[235] Liu H., Zhang Y., Zhang Q., Zhang T., Lu T. (2023). Metabolism-Related Prognostic Biomarkers, Purine Metabolism and Anti-Tumor Immunity in Colon Adenocarcinoma. Front. Biosci. (Landmark Ed.).

[236] Huang A., Sun Z., Hong H., Yang Y., Chen J., Gao Z., Gu J. (2024). Novel hypoxia- and lactate metabolism-related molecular subtyping and prognostic signature for colorectal cancer. J. Transl. Med..

[237] Ren Y., He S., Feng S., Yang W. (2022). A Prognostic Model for Colon Adenocarcinoma Patients Based on Ten Amino Acid Metabolism Related Genes. Front. Public Health.

[238] Sun T., Chen Y., Chen Y.X. (2025). Single-cell and bulk transcriptome analyses reveal elevated amino acid metabolism promoting tumor-directed immune evasion in colorectal cancer. Front. Immunol..

[239] Yue J., Fang H., Yang Q., Feng R., Ren G. (2025). Integrating multi-omics and machine learning methods reveals the metabolism of amino acids and derivatives-related signature in colorectal cancer. Front. Oncol..

[240] Zhang Y., Zhu H., Fan J., Zhao J., Xia Y., Zhang N., Xu H. (2025). A glutamine metabolism gene signature with prognostic and predictive value for colorectal cancer survival and immunotherapy response. Front. Mol. Biosci..

[241] Liu J., Li H., Wang L., Wang S., Tang Q. (2024). Spatial transcriptome and single-cell reveal the role of nucleotide metabolism in colorectal cancer progression and tumor microenvironment. J. Transl. Med..

[242] Kavran A.J., Bai Y., Rabe B., Kreshock A., Fisher A., Cheng Y., Lewin A., Dai C., Meyer M.J., Mavrakis K.J. (2025). Spatial genomics reveals cholesterol metabolism as a key factor in colorectal cancer immunotherapy resistance. Front. Oncol..

[243] Zhang W., Xia M., Li J., Liu G., Sun Y., Chen X., Zhong J. (2025). Warburg effect and lactylation in cancer: Mechanisms for chemoresistance. Mol. Med..

[244] Zhu W., Fan C., Hou Y., Zhang Y. (2025). Lactylation in tumor microenvironment and immunotherapy resistance: New mechanisms and challenges. Cancer Lett..

[245] Chu Y.-D., Cheng L.-C., Lim S.-N., Lai M.-W., Yeh C.-T., Lin W.-R. (2023). Aldolase B-driven lactagenesis and CEACAM6 activation promote cell renewal and chemoresistance in colorectal cancer through the Warburg effect. Cell Death Dis..

[246] Li W., Zhou C., Yu L., Hou Z., Liu H., Kong L., Xu Y., He J., Lan J., Ou Q. (2023). Tumor-derived lactate promotes resistance to bevacizumab treatment by facilitating autophagy enhancer protein RUBCNL expression through histone H3 lysine 18 lactylation (H3K18la) in colorectal cancer. Autophagy.

[247] Zhou Y., Tao Q., Luo C., Chen J., Chen G., Sun J. (2025). Epacadostat Overcomes Cetuximab Resistance in Colorectal Cancer by Targeting IDO-Mediated Tryptophan Metabolism. Cancer Sci..

[248] Li Y., Li C., Yao X., Lv J., Li W., Fu R., Chen M., Yang P., Dai Q., Wei W. (2024). IDO1-mediated kynurenine production inhibits IGFBP5 signaling to promote 5-fluorouracil-induced senescence escape and chemoresistance in colorectal cancer. Am. J. Cancer Res..

[249] Huang Y., Chan S., Chen S., Liu X., Li M., Zheng L., Dong Z., Yang Z., Liu Z., Zhou D. (2024). Wnt/β-catenin signalling activates IMPDH2-mediated purine metabolism to facilitate oxaliplatin resistance by inhibiting caspase-dependent apoptosis in colorectal cancer. J. Transl. Med..

[250] Li S., Fang W., Zheng J., Peng Z., Yu B., Chen C., Zhang Y., Jiang W., Yuan S., Zhang L. (2023). Whole-transcriptome defines novel glucose metabolic subtypes in colorectal cancer. J. Cell. Mol. Med..

[251] Huang H., Chen K., Zhu Y., Hu Z., Wang Y., Chen J., Li Y., Li D., Wei P. (2024). A multi-dimensional approach to unravel the intricacies of lactylation related signature for prognostic and therapeutic insight in colorectal cancer. J. Transl. Med..

[252] Zhao S., Zhang P., Niu S., Xie J., Liu Y., Liu Y., Zhao N., Cheng C., Lu P. (2024). Targeting nucleotide metabolic pathways in colorectal cancer by integrating scRNA-seq, spatial transcriptome, and bulk RNA-seq data. Funct. Integr. Genom..

[253] Xiao Y., Yu T.J., Xu Y., Ding R., Wang Y.P., Jiang Y.Z., Shao Z.M. (2023). Emerging therapies in cancer metabolism. Cell Metab..

[254] Pang B., Wu H. (2025). Metabolic reprogramming in colorectal cancer: A review of aerobic glycolysis and its therapeutic implications for targeted treatment strategies. Cell Death Discov..

[255] Zhong X., He X., Wang Y., Hu Z., Huang H., Zhao S., Wei P., Li D. (2022). Warburg effect in colorectal cancer: The emerging roles in tumor microenvironment and therapeutic implications. J. Hematol. Oncol..

[256] Akce M., Farran B., Switchenko J.M., Rupji M., Kang S., Khalil L., Ruggieri-Joyce A., Olson B., Shaib W.L., Wu C. (2023). Phase II trial of nivolumab and metformin in patients with treatment-refractory microsatellite stable metastatic colorectal cancer. J. Immunother. Cancer.

[257] Buckley C.E., O’bRien R.M., Nugent T.S., Donlon N.E., O’cOnnell F., Reynolds J.V., Hafeez A., O’rÍordáin D.S., Hannon R.A., Neary P. (2023). Metformin is a metabolic modulator and radiosensitiser in rectal cancer. Front. Oncol..

[258] Huang X., Sun T., Wang J., Hong X., Chen H., Yan T., Zhou C., Sun D., Yang C., Yu T. (2023). Metformin Reprograms Tryptophan Metabolism to Stimulate CD8+ T-cell Function in Colorectal Cancer. Cancer Res..

[259] Kang J., Lee D., Lee K.J., Yoon J.E., Kwon J.-H., Seo Y., Kim J., Chang S.Y., Park J., Kang E.A. (2022). Tumor-Suppressive Effect of Metformin via the Regulation of M2 Macrophages and Myeloid-Derived Suppressor Cells in the Tumor Microenvironment of Colorectal Cancer. Cancers.

[260] Broadfield L.A., Saigal A., Szamosi J.C., Hammill J.A., Bezverbnaya K., Wang D., Gautam J., Tsakiridis E.E., Di Pastena F., McNicol J. (2022). Metformin-induced reductions in tumor growth involves modulation of the gut microbiome. Mol. Metab..

[261] Gu X.-Y., Yang J.-L., Lai R., Zhou Z.-J., Tang D., Hu L., Zhao L.-J. (2025). Impact of lactate on immune cell function in the tumor microenvironment: Mechanisms and therapeutic perspectives. Front. Immunol..

[262] Rahman A., Yadab M.K., Ali M.M. (2024). Emerging Role of Extracellular pH in Tumor Microenvironment as a Therapeutic Target for Cancer Immunotherapy. Cells.

[263] Johnston R.J., Su L.J., Pinckney J., Critton D., Boyer E., Krishnakumar A., Corbett M., Rankin A.L., DiBella R., Campbell L. (2019). VISTA is an acidic pH-selective ligand for PSGL-1. Nature.

[264] Thisted T., Smith F.D., Mukherjee A., Kleschenko Y., Feng F., Jiang Z.-G., Eitas T., Malhotra K., Biesova Z., Onumajuru A. (2024). VISTA checkpoint inhibition by pH-selective antibody SNS-101 with optimized safety and pharmacokinetic profiles enhances PD-1 response. Nat. Commun..

[265] Oh M.-H., Sun I.-H., Zhao L., Leone R.D., Sun I.M., Xu W., Collins S.L., Tam A.J., Blosser R.L., Patel C.H. (2020). Targeting glutamine metabolism enhances tumor-specific immunity by modulating suppressive myeloid cells. J. Clin. Investig..

[266] Miyamoto R., Takigawa H., Yuge R., Shimizu D., Ariyoshi M., Otani R., Tsuboi A., Tanaka H., Yamashita K., Hiyama Y. (2024). Analysis of anti-tumor effect and mechanism of GLS1 inhibitor CB-839 in colorectal cancer using a stroma-abundant tumor model. Exp. Mol. Pathol..

[267] Ciombor K.K., Bae S.-W., Whisenant J.G., Ayers G.D., Sheng Q., Peterson T.E., Smith G.T., Lin K., Chowdhury S., Marie P.K. (2025). Results of the Phase I/II Study and Preliminary B-cell Gene Signature of Combined Inhibition of Glutamine Metabolism and EGFR in Colorectal Cancer. Clin. Cancer Res..

[268] Murata S., Yanagisawa K., Fukunaga K., Oda T., Kobayashi A., Sasaki R., Ohkohchi N. (2010). Fatty acid synthase inhibitor cerulenin suppresses liver metastasis of colon cancer in mice. Cancer Sci..

[269] Wang J., Deng S., Cheng D., Gu J., Qin L., Mao F., Xue Y., Jiang Z., Chen M., Zou F. (2024). Engineered microparticles modulate arginine metabolism to repolarize tumor-associated macrophages for refractory colorectal cancer treatment. J. Transl. Med..

[270] Yan Z., Wang B., Shen Y., Ren J., Chen M., Jiang Y., Wu H., Dai W., Zhang H., Wang X. (2025). Bisphosphonate-mineralized nano-IFNγ suppresses residual tumor growth caused by incomplete radiofrequency ablation through metabolically remodeling tumor-associated macrophages. Theranostics.

[271] Jang J.H., Kim J.-Y., Lee T.-J. (2024). Recent advances in anticancer mechanisms of molecular glue degraders: Focus on RBM39-dgrading synthetic sulfonamide such as indisulam, E7820, tasisulam, and chloroquinoxaline sulfonamide. Genes Genom..

[272] Ajab S.M., Zoughbor S.H., Labania L.A., Östlundh L.M., Orsud H.S., Olanda M.A., Alkaabi O., Alkuwaiti S.H., Alnuaimi S.M., Al Rasbi Z. (2024). Microbiota composition effect on immunotherapy outcomes in colorectal cancer patients: A systematic review. PLoS ONE.

[273] Neagu A.I., Bostan M., Ionescu V.A., Gheorghe G., Hotnog C.M., Roman V., Mihaila M., Stoica S.I., Diaconu C.C., Diaconu C.C. (2025). The Impact of the Microbiota on the Immune Response Modulation in Colorectal Cancer. Biomolecules.

[274] Jiang S.-S., Xie Y.-L., Xiao X.-Y., Kang Z.-R., Lin X.-L., Zhang L., Li C.-S., Qian Y., Xu P.-P., Leng X.-X. (2023). *Fusobacterium nucleatum*-derived succinic acid induces tumor resistance to immunotherapy in colorectal cancer. Cell Host Microbe.

[275] Yu L., Guo Q., Gu X., Wang Z., Li J., Wang X., Xu Z., Wang Y., Zhang Y., Zhang Y. (2025). Impact of gut microbiome on radiotherapy and immunotherapy efficacy in microsatellite-stable colorectal cancer: Role of propionic acid and *B. fragilis*. Br. J. Cancer.

[276] Luo M., Wang Q., Sun Y., Jiang Y., Wang Q., Gu Y., Hu Z., Chen Q., Xu J., Chen S. (2024). Fasting-mimicking diet remodels gut microbiota and suppresses colorectal cancer progression. npj Biofilms Microbiomes.

[277] Nan K., Zhong Z., Yue Y., Shen Y., Zhang H., Wang Z., Zhuma K., Yu B., Fu Y., Wang L. (2025). Fasting-mimicking diet-enriched *Bifidobacterium pseudolongum* suppresses colorectal cancer by inducing memory CD8 ^+^ T cells. Gut.

[278] Zhong Z., Zhang H., Nan K., Zhong J., Wu Q., Lu L., Yue Y., Zhang Z., Guo M., Wang Z. (2023). Fasting-Mimicking Diet Drives Antitumor Immunity against Colorectal Cancer by Reducing IgA-Producing Cells. Cancer Res..

[279] Liu X., Peng S., Tang G., Xu G., Xie Y., Shen D., Zhu M., Huang Y., Wang X., Yu H. (2023). Fasting-mimicking diet synergizes with ferroptosis against quiescent, chemotherapy-resistant cells. eBioMedicine.

[280] Xu H., Wang Y., Liu G., Zhu Z., Shahbazi M., Reis R.L., Kundu S.C., Shi X., Zu M., Xiao B. (2024). Nano-Armed *Limosilactobacillus reuteri* for Enhanced Photo-Immunotherapy and Microbiota Tryptophan Metabolism against Colorectal Cancer. Adv. Sci..

[281] Zhang M., Chen G., Jin X., Wang J., Yu S. (2024). Pre-Operative Immunonutrition Enhances Postoperative Outcomes and Elevates Tumor-Infiltrating Lymphocyte Counts in Colorectal Cancer Patients: A Meta-Analysis of Randomized Controlled Trials. Nutr. Cancer.

[282] Xu J., Sun X., Xin Q., Cheng Y., Zhan Z., Zhang J., Wu J. (2018). Effect of immunonutrition on colorectal cancer patients undergoing surgery: A meta-analysis. Int. J. Color. Dis..

[283] Ambrosio M.R., Spagnoli L., Perotti B., Petrelli F., Caini S., Saieva C., Usai S., Bianchini M., Cavazzana A., Arganini M. (2023). Paving the Path for Immune Enhancing Nutrition in Colon Cancer: Modulation of Tumor Microenvironment and Optimization of Outcomes and Costs. Cancers.

[284] Dmitrieva-Posocco O., Wong A.C., Lundgren P., Golos A.M., Descamps H.C., Dohnalová L., Cramer Z., Tian Y., Yueh B., Eskiocak O. (2022). β-Hydroxybutyrate suppresses colorectal cancer. Nature.

[285] Li Z., Zhang S., Zhang Y., Chen J., Wu F., Liu G., Chen G. (2021). Applications and Mechanism of 3-Hydroxybutyrate (3HB) for Prevention of Colonic Inflammation and Carcinogenesis as a Food Supplement. Mol. Nutr. Food Res..

[286] Fan X., Zhu M., Qiu F., Li W., Wang M., Guo Y., Xi X., Du B. (2020). Curcumin may be a potential adjuvant treatment drug for colon cancer by targeting CD44. Int. Immunopharmacol..

[287] Jamialahmadi T., Guest P.C., Tasbandi A., Majeed M., Sahebkar A. (2022). Testing the Anti-inflammatory Effects of Curcuminoids in Patients with Colorectal Cancer. Methods Mol. Biol..

[288] Kalinski T., Sel S., Hütten H., Röpke M., Roessner A., Nass N. (2014). Curcumin Blocks Interleukin-1 Signaling in Chondrosarcoma Cells. PLoS ONE.

[289] Peschel D., Koerting R., Nass N. (2007). Curcumin induces changes in expression of genes involved in cholesterol homeostasis. J. Nutr. Biochem..

[290] Shih K.-C., Chan H.-W., Wu C.-Y., Chuang H.-Y. (2023). Curcumin Enhances the Abscopal Effect in Mice with Colorectal Cancer by Acting as an Immunomodulator. Pharmaceutics.

[291] Feltrin F.d.S., Agner T., Sayer C., Lona L.M.F. (2022). Curcumin encapsulation in functional PLGA nanoparticles: A promising strategy for cancer therapies. Adv. Colloid Interface Sci..

[292] Feng T., Wei Y., Lee R.J., Zhao L. (2017). Liposomal curcumin and its application in cancer. Int. J. Nanomed..

[293] Zhu X., Yu Z., Feng L., Deng L., Fang Z., Liu Z., Li Y., Wu X., Qin L., Guo R. (2021). Chitosan-based nanoparticle co-delivery of docetaxel and curcumin ameliorates anti-tumor chemoimmunotherapy in lung cancer. Carbohydr. Polym..

[294] Deshmukh R., Prajapati M., Harwansh R.K. (2024). Management of Colorectal Cancer Using Nanocarriers-based Drug Delivery for Herbal Bioactives: Current and Emerging Approaches. Curr. Pharm. Biotechnol..

[295] Roy A., Raza M.A., Ghosh V., Ajazuddin (2024). Diagnostic innovations and therapeutic potential of nanoparticulate delivery for colon cancer. Nano-Struct. Nano-Objects.

